# Post-COVID exercise intolerance is associated with capillary alterations and immune dysregulations in skeletal muscles

**DOI:** 10.1186/s40478-023-01662-2

**Published:** 2023-12-08

**Authors:** Tom Aschman, Emanuel Wyler, Oliver Baum, Andreas Hentschel, Rebekka Rust, Franziska Legler, Corinna Preusse, Lil Meyer-Arndt, Ivana Büttnerova, Alexandra Förster, Derya Cengiz, Luiz Gustavo Teixeira Alves, Julia Schneider, Claudia Kedor, Judith Bellmann-Strobl, Aminaa Sanchin, Hans-Hilmar Goebel, Markus Landthaler, Victor Corman, Andreas Roos, Frank L. Heppner, Helena Radbruch, Friedemann Paul, Carmen Scheibenbogen, Nora F. Dengler, Werner Stenzel

**Affiliations:** 1 Department of Neuropathology, Charité - Universitätsmedizin Berlin, corporate member of Freie Universität Berlin, Humboldt Universität zu Berlin, Berlin Institute of Health, Berlin, Germany; 2 Max Delbrück Center for Molecular Medicine, Berlin Institute for Medical Systems Biology, Berlin, Germany; 3 Institute of Physiology, Charité - Universitätsmedizin Berlin, corporate member of Freie Universität Berlin, Humboldt Universität zu Berlin, Berlin Institute of Health, Berlin, Germany; 4 Leibniz-Institut Für Analytische Wissenschaften - ISAS - E.V, Dortmund, Germany; 5 Experimental and Clinical Research Center and NeuroCure Clinical Research Center, Charité - Universitätsmedizin Berlin, corporate member of Freie Universität Berlin, Humboldt Universität zu Berlin, Berlin Institute of Health, Berlin, Germany; 6 Department of Neurology, Charité - Universitätsmedizin Berlin, corporate member of Freie Universität Berlin, Humboldt Universität zu Berlin, Berlin Institute of Health, Berlin, Germany; 7 Department of Autoimmune Diagnostics, Labor Berlin-Charité Vivantes GmbH, Berlin, Germany; 8 Department of Neurology, Medical Faculty, Heinrich-Heine-University, Düsseldorf, Germany; 9 Institute of Virology, Charité - Universitätsmedizin Berlin, corporate member of Freie Universität Berlin, Humboldt Universität zu Berlin, Berlin Institute of Health, Berlin, Germany; 10 Institute of Medical Immunology, Charité - Universitätsmedizin Berlin, corporate member of Freie Universität Berlin, Humboldt Universität zu Berlin, Berlin Institute of Health, Berlin, Germany; 11 Department of Neurosurgery, Charité - Universitätsmedizin Berlin, corporate member of Freie Universität Berlin, Humboldt Universität zu Berlin, Berlin Institute of Health, Berlin, Germany; 12 Department of Neuropathology, Universitätsmedizin Mainz, Mainz, Germany; 13 Institute for Biology, Humboldt-Universität zu Berlin, Berlin, Germany; 14 Department of Pediatric Neurology, Faculty of Medicine, University Children’s Hospital, University of Duisburg-Essen, Essen, Germany; 15 Department of Neurology Bergmannsheil, Heimer-Institut Für Muskelforschung am Bergmannsheil, Bochum, Germany; 16 Cluster of Excellence, NeuroCure, Berlin, Germany; 17 German Center for Neurodegenerative Diseases (DZNE) Berlin, Berlin, Germany; 18 Max Delbrueck Center for Molecular Medicine, Berlin, Germany

**Keywords:** SARS-CoV-2, Post-COVID syndrome, Post-acute sequelae of COVID-19 (PASC), Post-infectious syndrome, Microangiopathy, Basement membrane thickening, ME/CFS

## Abstract

The SARS-CoV-2 pandemic not only resulted in millions of acute infections worldwide, but also in many cases of post-infectious syndromes, colloquially referred to as “long COVID”. Due to the heterogeneous nature of symptoms and scarcity of available tissue samples, little is known about the underlying mechanisms. We present an in-depth analysis of skeletal muscle biopsies obtained from eleven patients suffering from enduring fatigue and post-exertional malaise after an infection with SARS-CoV-2. Compared to two independent historical control cohorts, patients with post-COVID exertion intolerance had fewer capillaries, thicker capillary basement membranes and increased numbers of CD169^+^ macrophages. SARS-CoV-2 RNA could not be detected in the muscle tissues. In addition, complement system related proteins were more abundant in the serum of patients with PCS, matching observations on the transcriptomic level in the muscle tissue. We hypothesize that the initial viral infection may have caused immune-mediated structural changes of the microvasculature, potentially explaining the exercise-dependent fatigue and muscle pain.

**Graphical Abstract:**

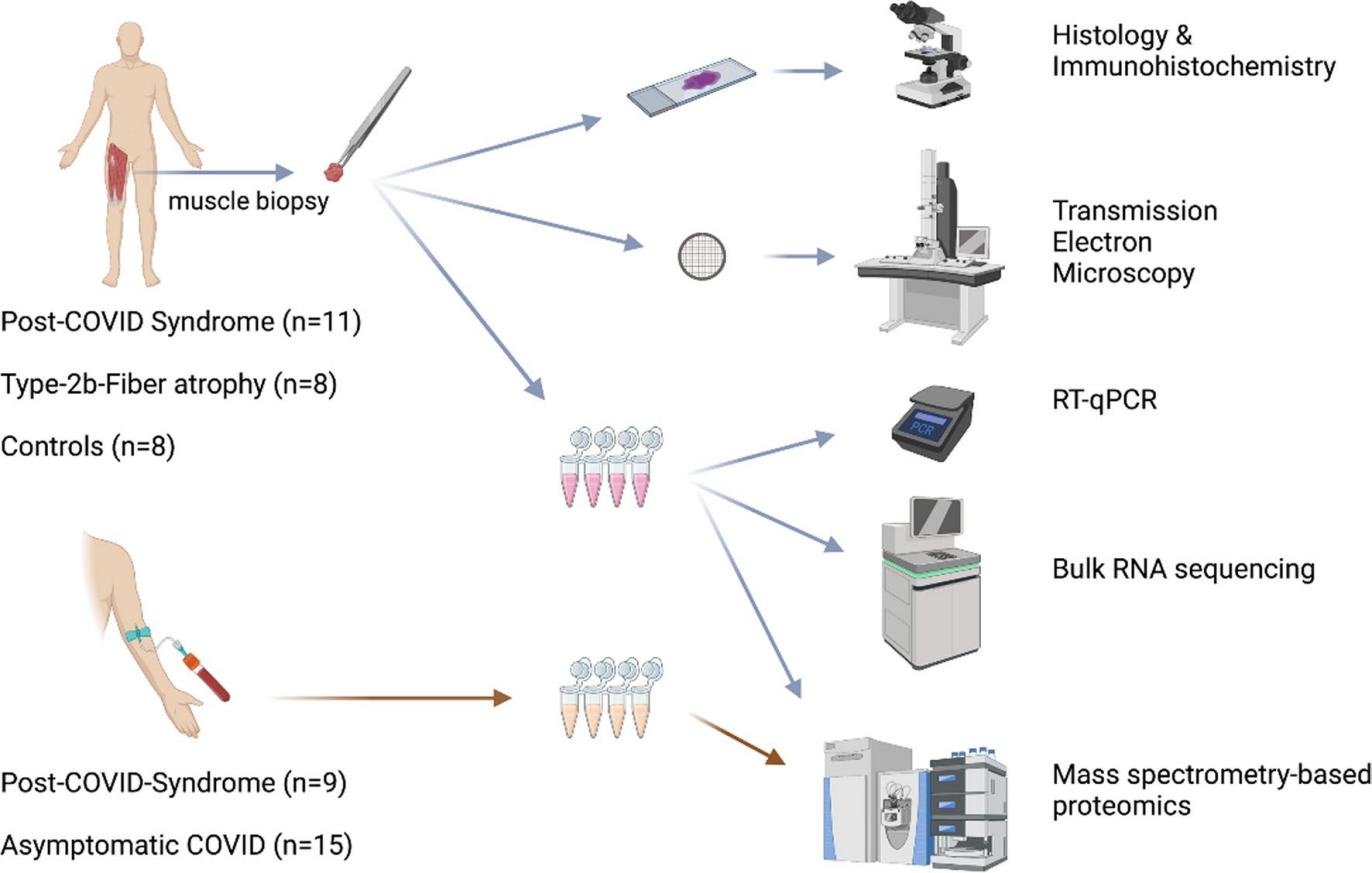

**Supplementary Information:**

The online version contains supplementary material available at 10.1186/s40478-023-01662-2.

## Introduction

Persisting general or muscular fatigue are well-known phenomena occurring in a proportion of previously healthy individuals after an acute viral infection, and many endemic or epidemic outbreaks of such symptoms have been documented [1–5]. While most people infected with SARS-CoV-2 recover without sequelae, subsets of both outpatient and hospitalized individuals experience newly occurring and lasting symptoms such as fatigue, muscular weakness, exercise intolerance with post-exertional malaise (PEM), myalgia or neurocognitive and neuropsychiatric disturbances [6–11]. The pathomechanisms behind *post-COVID Syndrome* (PCS), *Post-acute sequelae of SARS-CoV-2 infection* or *Long COVID*, are not well understood, plausibly because the manifestations and causes are diverse and semantically diffuse, thus hampering comparative descriptive and mechanistic studies. Similar to post-SARS-syndrome reported after the SARS epidemic in 2003–2004 [12, 13], many clinicians noticed that some prevailing symptoms of PCS were reminiscent of chronic fatigue syndrome (ME/CFS) [14–17]. This syndrome is based on clinical criteria and exclusion of other diagnoses, allegedly consisting of different causal entities resulting in a similar phenotype, centered around a chronic fatigue aggravated by physical and/or mental activity and accompanied by other symptoms (e.g. sleep disturbances, neurocognitive deficits), leading to significant restrictions in everyday life [17–21]. In most cases, ME/CFS is triggered by an infection [22–28].

While inflammatory myopathy has been shown in individuals with fatal COVID-19 [29, 30], to our knowledge no case–control studies investigated the affection of skeletal muscle tissue in individuals with PCS. We therefore carried out this study of vastus lateralis muscle biopsy samples obtained from individuals suffering from chronic muscular fatigue, myalgia and PEM after a PCR-proven infection with SARS-CoV-2. Biopsies were taken in average one year after the initial infection and were compared to historical control samples, consisting of histologically normal samples as well as samples with a selective atrophy of type-2b muscle fibers. The latter is a non-specific finding associated with muscle disuse and immobility. While patients with PCS did not show overt signs of myositis, increased numbers of CD169^+^ macrophages were evident. The capillary-to-fiber ratio was decreased and ultrastructural analysis revealed a capillary basement membrane enlargement. SARS-CoV-2-specific RNA was not detected, but transcriptomic analysis highlighted increased expressions of genes related to immune system regulation (complement system, type I and III interferons, macrophage markers), angiogenesis and extracellular matrix remodeling, and decreased expressions of genes related to metabolic processes and mitochondrial activity. Proteome analysis of the serum revealed a higher abundance of complement pathway-related proteins compared to sera of people that fully recovered after a SARS-CoV-2-infection.

## Results

### Clinical characteristics of the post-COVID syndrome cohort

**Fig. 1:**
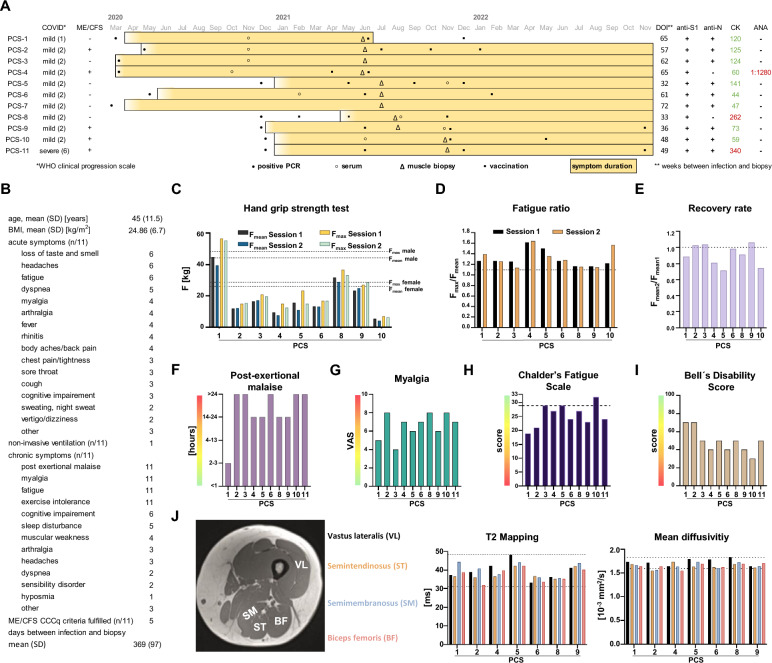
Clinical characteristics of the post-COVID syndrome cohort. Demographics of study participants with Post-COVID Syndrome (PCS) and timeline of events are shown in (**A**). Black dots indicate the month in which PCR-testing for SARS-CoV-2 was positive. Black circles indicate the month from which serum samples were available. For two patients (*PCS-7* and *PCS-11*), no serum was available for mass spectrometry and myositis-specific and –associated antibody screening. Black triangles indicate the month in which biopsy of vastus lateralis muscle was performed. Black cubes indicate the months of anti-SARS-CoV-2 vaccinations. Colored bars indicate the months in which the participants reported symptoms. Patients characteristics, summary of acute and chronic symptoms are reported in (**B**). Handgrip strength test (HGS) was performed in two subsequent sessions (session 1 and 2) in 9/11 patients around the time of biopsy (**C**). Dotted lines indicate the mean reference values based on ROC analyses showing the highest sensitivity and specifity to discriminate between healthy controls and ME/CFS patients for men and women respectively (Jakel et al. 2021). Fatigue ratio defined as the ratio of F_max_ / F_mean_ per individual session (**D**). Dotted lines indicate the respective normal population mean reference values for women and men (Jakel et al. 2021). Recovery rate defined as the ratio of F _mean_ of session 2 divided by F _mean_ of session 1 (**E**). Dotted lines indicate the normal population mean reference value (Jakel et al. 2021). Duration of post-exertional malaise around the time of biopsy (**F**). Intensity of myalgia on the VAS (Visual analog scale), 0 = no muscle pain; 10 = highest pain imagineable) (**G**). Chalder`s Fatigue scale around the time of biopsy (**H**) Dotted lines indicate reference values for the general population. Bell disability score around the time of biopsy (**I**). MRI of proximal lower extremity with indication of ROI (regions of interest) and results of T2 mapping and mean diffusivity (**J**). *Abbreviations: HDC* Healthy-diseased control, *PCS* Post-COVID syndrome, *2BA* type-2b atrophy control, *BMI* Body mass index, *SD* Standard deviation, *ME/CFS* Myalgic encephalomyelitis/chronic fatigue syndrome, *DOI* Duration of illness (at timepoint of biopsy), *CK* Creatine kinase, *ANA* Anti-nuclear antibodies, *F* Force

Eleven individuals (female-to-male ratio 10:1; mean age 45.1 years, [SD 11.5]; range 25–58 years) who were tested positive for SARS-CoV-2 by PCR between March 2020 and December 2020, and who subsequently developed a new fatigue with muscular symptoms (weakness, myalgia) and exertion intolerance with post-exertional malaise (PEM) lasting for at least 6 months which could not be explained by alternative diagnoses underwent a biopsy of vastus lateralis muscle, fascia and skin. Nine of these patients were part of a larger observational study during which they were asked to submit subjective symptom surveys at defined intervals [14]. The majority of patients (91%; *n* = 10/11) only had mild COVID as defined by the WHO [31]. One patient however (*PCS-11*) was hospitalized and required four days of non-invasive ventilation due to dyspnea, thus reaching a score of 6 (severe COVID) [31]. The timeline of events, as well as the documented acute and chronic symptoms are reported in Fig. 1A and B and Additional file 1: Table S1.

All patients complained of persisting fatigue with exercise intolerance and myalgia at the time of study inclusion. In all but one patient, symptoms were still present by the end of November 2022 and five patients fulfilled the Canadian Consensus Criteria (CCC) for ME/CFS [32]. The average time between onset of acute infection symptoms or positive PCR and muscle biopsy was 369 days (SD 97) (Fig. 1A and B).

Standardized handgrip strength test (HGS) was performed in nine, and a 6-min-walk-test (6MWT) in ten patients. Compared to reference values [33], seven patients (78%; *n* = 7/9) showed abnormal values for maximal and mean force, fatigue ratio and recovery rate in the HGS (Fig. 1C–E). Seven patients (70%; *n* = 7/10) walked a shorter distance within six minutes than expected for their age and sex [34] (Additional file 1: Fig. S1A). Most patients reported a PEM of at least 14 h (Fig. 1F). Perceived symptoms and impairments were assessed by standardized questionnaires, including the *Bell disability score*^1^* (0–100; 0* = *bedridden, 100* = *healthy)*, *Chalder fatigue score* [35], *SF-36* [36], *EQ-5D-5L* [37, 38] and *Zung Self-Rating Depression Scale* [39]. Ten individuals (91%) scored 50 or lower on the Bell disability score, indicating a functionality level below 70% compared to the German reference population (Fig. 1I). The mean individual health related quality of life on the visual analog scale from 0 to 100 in our cohort was 50.8 (SD 18.7), being substantially lower than the average German population which was shown to be 79.5 (SD 17.1) [38] (Additional file 1: Fig. S1A). The EQ-5D-5L mean scores for the dimensions *mobility*, *self-care*, *usual activities*, *pain* and *anxiety* on a scale from 1 (no problems) to 5 (extreme problems) were 3/2/4/4/2, indicating moderate affection in the dimensions *mobility* and severe problems within the dimensions *usual activities* and *pain*, respectively. Most of these dimension’s scores as well as other functional scores did not evolve for the better in some patients (*PCS-2, PCS-3, PCS-4, PCS-6, PCS-10*) while they improved to some degree in others (*PCS-1, PCS-5, PCS-7, PCS-8, PCS-9, PCS-11*) (Additional file 1: Fig. S1B).

Proximal lower extremity MRI was performed in nine patients on the same day or close to the day of biopsy. This revealed no radiological evidence for myositis in the biceps femoris (BF), semitendinosus (ST), semimembranosus (SM), and vastus lateralis (VL) muscles. T2 mapping and mean diffusivity were comparable to levels of healthy control cohorts from published studies [40–43] (Fig. 1J). Muscle Fat Fraction (MFF), depending on age, physical fitness, sex and hormonal components were comparable to reference values in most subjects, but two study participants (*PCS-6* and *PCS-9*) showed slightly increased values in two of the four examined muscles [44, 45] (Additional file 1: Fig. S1E). Comparability to published data is however limited due to technical differences in acquisition.

Heart MRI revealed a discrete pericarditis in one patient (*PCS-4*), that had disappeared in a follow-up examination 8 months later. Diffuse fibrotic changes in one other patient (*PCS-5*) and a dilated cardiomyopathy in yet another patient (*PCS-11*) were noted (Additional file 1: Table S1). Levels of creatine kinase were below the lab threshold (< 167 U/L) in all but two patients, who only had slightly increased levels (*PCS-8*: 1.5-fold increase; *PCS-11*: twofold increase). One patient (*PCS-4*) had high titers of antinuclear antibodies (1:1280) with a dense fine speckled nuclear staining pattern, without detection of specific autoantibodies (Fig. 1A). Screening for myositis-specific and myositis-associated autoantibodies revealed the presence of anti-PM-Scl75 antibodies in two patients, one of them also having anti-PL-12 antibodies, and anti-PM-Scl100 antibodies in another patient (Additional file 1: Fig. S2).

### Confirmation of SARS-CoV-2 infection in the PCS cohort and absence of virus detection in skeletal muscle specimen

All patients from the PCS cohort were tested positive for SARS-CoV-2 by PCR from nasopharyngeal swabs in the course of 2020. Presence and titers of antibodies against SARS-CoV-2 spike (S) and nucleocapsid (N) antigen were determined using two different techniques (ELISA and *ECLIA).* This revealed a presence of anti-S-IgG in all patients and of anti-N-IgG in 9/11 patients. One of the two patients without anti-N-IgG had detectable anti-S-IgG above the positivity threshold already prior to vaccination, also proving a contact with SARS-CoV-2 (Fig. 1A, Additional file 1: Fig. S1C).

Quantitative reverse transcription–polymerase chain reaction (RT-qPCR) of skeletal muscle specimen did not yield SARS-CoV-2 specific RNA amplification below the lab threshold cycle. In one sample (*PCS-4*), a first reaction gave a weakly positive result that could not be confirmed in a repeat experiment.

### Type-2b-fiber atrophy and increased numbers of tissue macrophages in skeletal muscles of patients with PCS

**Fig. 2:**
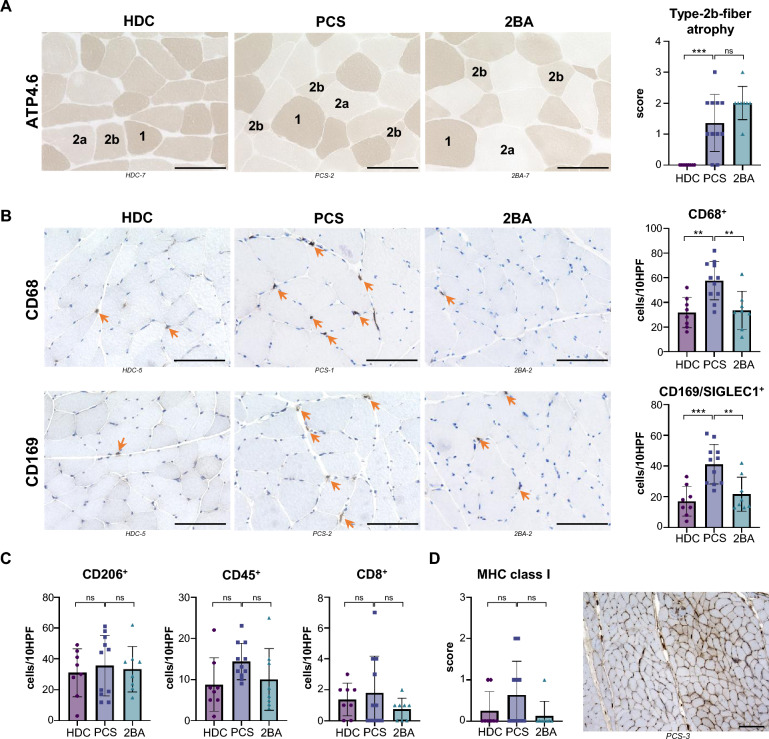
Type-2b-fiber atrophy and increased numbers of tissue macrophages in skeletal muscles of patients with PCS. ATP4.6 enzymatic reaction showing selective atrophy of type-2b muscle fibers in PCS samples and the 2BA control cohort (**A**). Immunohistochemistry and quantification of CD68^+^ and CD169^+^ macrophages (**B**). Immunohistochemistry and quantification of CD206^+^, CD45^+^ and CD8^+^ cells (**C**). 400 × magnification, scale bar = 100 µm. Immunohistochemistry, semi-quantitative scoring of sarcolemmal MHC-cl.-I expression and example with a score of 2 (**D**). 100 × magnification, black scale bar = 200 µm. Ordinary one-way ANOVA with Tukey's multiple comparisons test. * = *p* < 0.05, ** = *p* < 0.005, *** = *p* < 0.0005. *ATP4.6* Adenosine 5'-TriPhosphatase at pH 4.6, *SIGLEC1* Sialoadhesin, *MHC cl. I* Major histocompatibility complex class I

Routine histopathological examination revealed a selective atrophy of type-2b muscle fibers of differing extent in 72% (*n* = 8/11) of individuals with PCS and – by definition of our inclusion criteria – in none (*n* = 0/8) of the HDC cohort and in all (*n* = 8/8) of the 2BA cohort (Fig. 2A). Fiber type composition (percentages of type 1 and type 2 fibers) were comparable between the 3 cohorts (Additional file 1: Fig. S3E). No sarcolemmal expression of C5b-9 was found in any sample, but in one patient from the PCS cohort (*PCS-11*) a weak capillary expression of C5b-9 was present (Additional file 1: Fig. S3B). Single muscle fibers with sarcolemmal upregulation of MHC-class-I were found in two samples from the HDC cohort, in one from the 2BA and in three from the PCS cohort. These were judged as within normal range and not indicative of myositis. Two samples from the PCS cohort (*PCS-3* and *-10*) but none from the two control cohorts revealed a significant upregulation of MHC-class-I in at least 20% of the muscle fibers and if present, those fibers were not located in perifascicular regions (Fig. 2D). In one of these patients (*PCS-3*) a sarcolemmal upregulation of MHC-class-II was also present on some fibers (Additional file 1: Fig. S3C). Upregulation of Interferon-induced GTP-binding protein Mx1 was observed on several capillaries in one sample from the PCS cohort (*PCS-6*), but in none of the controls (Additional file 1: Fig. S3D).

Quantification of immune cells revealed a significant increase of CD68^+^ (*HDC vs. PCS*: mean of 32 [SD 12] versus 58 [SD 16] cells/10HPF, *p* = 0.002; *2BA vs. PCS*: mean of 34 [SD 15] versus 58 [SD 16] cells/10HPF, *p* = 0.005) and CD169^+^ (*HDC vs. PCS*: mean of 17 [SD 10] versus 41 [SD 13] cells/10HPF, *p* = 0.0004; *2BA vs. PCS*: mean of 22 [SD 11] versus 41 [SD 13] cells/10HPF, *p* = 0.004) macrophages. Numbers of CD45^+^ cells, CD8^+^ T-cells and CD206^+^ macrophages were not significantly different between the three groups (Fig. 2B and C).

### Decreased capillary-to-fiber ratio and increased capillary basement membrane thickness in patients with PCS

**Fig. 3:**
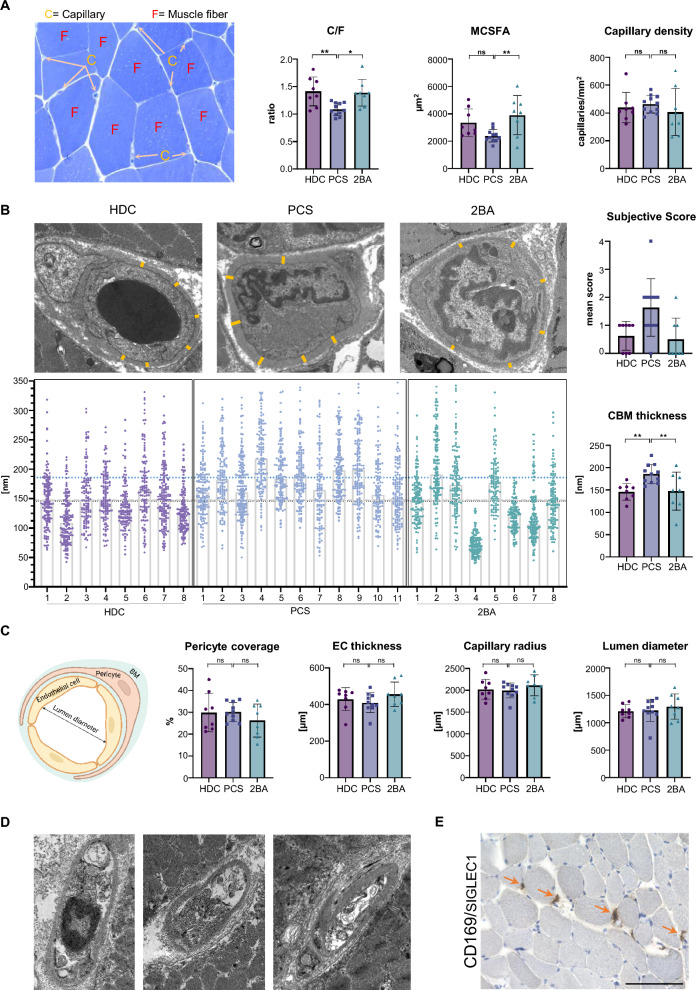
Decreased capillary-to-fiber ratio and thicker capillary basement membranes in patients with PCS. Example of a semithin transverse section of a vastus lateralis muscle biopsy specimen stained with toluidine blue, displaying capillaries and muscle fibers, and quantification of C/F, MCSFA and capillary density (**A**). Representative electron micrographs of transverse ultra-thin sections of muscle capillaries (**B**). Orange lines indicate typical sites of measurements for the capillary basement membrane thickness. The large graphs depicts all 3732 individual measurements except 35 outliers with values above 350 µm. Coloured dotted lines indicate the respective group means. Small graphs indicate subjective scoring of capillary pathology as well as mean CBM thickness values per individual samples. Schematic cross-sectional representation of a capillary (created with BioRender.com) with results of TBIA morphometric analysis (**C**). Exemplary micrographs of transverse ultra-thin sections of damaged muscular capillaries from *PCS-4* (**D**). Immunohistochemical expression of CD169/SIGLEC1 in the biopsy specimen obtained from *PCS-4* (**E**). 400 × magnification, black scale bar = 100 µm. Ordinary one-way ANOVA with Tukey's multiple comparisons test. * = *p* < 0.05, ** = *p* < 0.005, *** = *p* < 0,0005. *C/F* capillary-to-fiber ratio, *MCSFA* Mean cross-sectional fiber area, *CBM* Capillary basement membrane, *EC* Endothelial cell, *TBIA* Tablet-based image analysis

Numbers of capillaries per muscle fiber (C/F-ratio) were quantified by assessing at least 10 images of semithin cross-sections of all included muscle specimens at 200 × magnification in a blinded fashion. This revealed a significantly lower C/F-ratio in the PCS cohort when compared to the two historical control cohorts (*PCS vs. HDC*: mean difference 0.32, *p* = 0.007; *PCS vs. 2BA*: mean difference 0.30, *p* = 0.013). Comparison of mean cross-sectional fiber area (MCSFA) revealed a significant decrease in the PCS cohort compared to the 2BA cohort (mean difference 1508 µm^2^, *p* = 0.009) whereas no significant difference was present when compared to the HDC cohort. The significantly lower MCSFA in comparison to the 2BA cohort reflects an overall reduced fiber size in patients with PCS not only concerning type-2b-fibers (Fig. 3A).

The capillary density, which represents the absolute number of capillaries per mm^2^ of muscle area, was not significantly different between the three study groups (Fig. 3A). This seemingly counterintuitive result can be explained by the inverse correlation of the capillarity density (absolute number of capillaries per mm^2^) and the mean cross-sectional fiber area (MCSFA). In fact, a lower MCSFA results in more muscle fiber profiles and thus more capillary profiles within a field of vision, leading to higher absolute values for capillary density than C/F-ratio. We therefore consider the interpretation of the C/F to be more conclusive: although the capillary perfusion per volume of muscle was not different, the capillary supply of individual fibers was lower in muscles of PCS patients.

Between 20 and 30 capillaries per sample were photographed by TEM at a magnification of 7000x (mean number of capillaries analyzed per group: *HDC* 23.63 (SD 4), *2BA* 22.75 (SD 2) and *PCS* 24.27 (SD 3). For each individual capillary, the thickness of capillary basement membrane (CBM) was measured at six different sites, excluding areas with profiles of pericyte processes as the variation of CBM is more pronounced there [46]. This resulted in a total of 3732 single CBM measurements (Fig. 3B). CBM thickness was significantly increased in the PCS cohort (*PCS vs. HDC*: mean difference 39.99 µm, *p* = 0.016; *PCS vs. 2BA*: mean difference 38.48, *p* = 0.021). When excluding the apparent outlier from the 2BA cohort (*2BA-4*) the threshold for significance in that comparison was just not reached (*PCS vs. 2BA*: mean difference 28.54 µm, *p* = 0.06) (Fig. 3B).

Morphometry by TBIA revealed no significantly different values for lumen diameters and capillary radius between the groups, confirming that the capillaries that were selected for CBM thickness measurements were comparable in size and caliber. Furthermore, no difference regarding pericyte coverage and endothelial cell thickness were observed (Fig. 3C).

In one patient (*PCS-4*), massive structural damage was observed in all 23 photographed capillary profiles. Endothelial cells were almost completely degenerated, resulting in debris-containing empty capillary tubes also called string vessels (= acellular capillary remnants) (Fig. 3D). Such severe morphological alterations were not found in any other patient. Immunohistochemistry revealed the presence of many large CD169^+^ macrophages in close proximity to the capillaries (Fig. 3E).

There was a moderate positive correlation between the CBM thickness and the number of CD169^+^ macrophages (Pearson's r 0.53), between CBM thickness and the EQ-5D-5L mobility score (Pearson's r 0.78) and between CBM thickness and the EQ-5D-5L usual activities score around the time of biopsy (Pearson's r 0.56), but CBM thickness did not correlate with age (Additional file 1: Fig. S5A, B, D). There was also a correlation between the mean cross sectional fiber size area (MCSFA) and the capillary-to-fiber ratio (Pearson's r 0.63) (Additional file 1: Fig. S5C). These findings fit previous reports of a positive correlation between capillary numbers surrounding a muscle fiber and the diameter of that fiber [47].

### Increased abundance of basement membrane proteins in muscles from patients with PCS

**Fig. 4:**
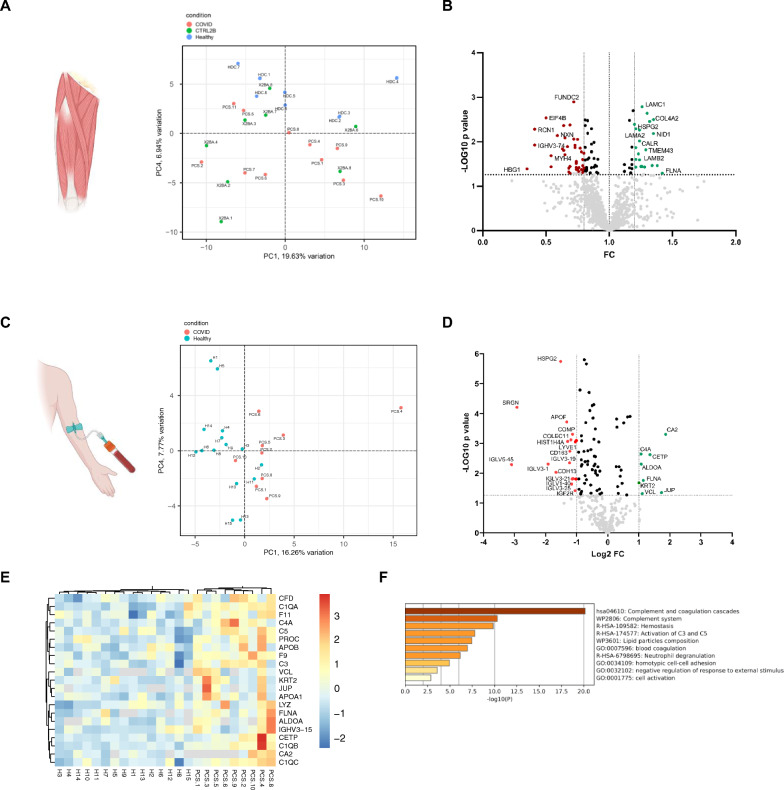
Increased expression of basement membrane proteins in muscles from patients with PCS. Principal component analysis of skeletal muscle proteome raw data after normalization (**A**). Volcano plot of the vastus lateralis proteome comparison illustrating significantly differentially abundant proteins between the PCS and the HDC cohort (**B**). The -log10 (p value) is plotted against the fold change of PCS/HDC. The dotted vertical lines denote + 1.2- and − 0.8-fold change respectively, while the dotted horizontal line denotes *p* = 0.05. Principal component analysis of serum proteome raw data after normalization (**C**). Volcano plot of the serum proteome comparison illustrating significantly differentially abundant proteins between the PCS (*n* = 9) and asymptomatic controls after an SARS-CoV-2-infection (*n* = 15) (**D**). The -log10 (p value) is plotted against the log twofold change of PCS/Controls. The dotted vertical lines denote + 1 and -1-log2-fold change respectively, while the dotted horizontal line denotes *p* = 0.05. Heatmap visualization of proteins with significantly increased abundance and their respective expression levels per individual sample (**E**). Metascape pathway enrichement analysis results from significantly upregulated proteins in the serum (**F**). Illustrations created with BioRender.com

Unbiased principal component analysis of mass spectrometric measurements from muscle specimen resulted in a clear separation of the HDC from the 2BA and PCS samples, while a less clear separation of the PCS patients from the 2BA samples could be observed (Fig. 4A). When comparing muscle specimen of patients with PCS to the HDC cohort, significantly higher levels of mostly matrisome-associated proteins related to or constituting basement membranes (COL4A2, NID1, HSPG2, LAMC1, LAMA2, LAMB2, FLNA) or muscle fibers (MYH2, SGCB) were found, albeit below the threshold of twofold change (Fig. 4B). Of interest, most of these proteins also had significantly higher expression levels when comparing samples from patients with PCS to samples from our 2BA cohort (Additional file 1: Fig. S4A and B). This matches our observation described above, that the mean values of CBM thickness in the 2BA cohort were closer to the PCS cohort than to the HDC cohort. Other notable proteins with increased expression levels in the PCS cohort are TAGLN2 and CALR. Four proteins were significantly decreased below a threshold of 0.5 (HBG1, IGHV3-74, RCN1 and EIF4B). Quantitative qRT-PCR did not reveal statistically significant differences in the gene expressions of CBM key components except for NID1 (Additional file 1: Fig. S4C). GO-Enrichment analysis revealed an upregulation of biological activity terms related to regulation of basement membrane and extracellular matrix (ECM) organization, as well as cell–cell and cell-ECM signaling (Additional file 1: Fig. S4D).

### Increased abundance of complement and coagulation cascade related proteins in the sera of individuals with PCS

Sera from patients with PCS (*n* = 9/11) were compared to sera from age- and sex-matched individuals that fully recovered from a SARS-CoV-2-infection. Principal component analysis of the normalized mass spectrometric raw data showed a moderate to clear separation of the two cohorts (Fig. 4C). An increased abundance of circulating proteins involved in the complement (CFD, C1QA, C1QB, C1QC, C4A, C5) and coagulation (procoagulant F11, F9, anticoagulant PROC) cascades were found in patients with PCS. Other proteins with increased abundance in that cohort play a role in cell–cell and cell–matrix adhesion (VCL, FLNA, JUP) and lipid transportation (APOB, APOA1, CETP) (Fig. 4 D–F).

### Distinct transcriptomic profiles of vastus lateralis muscles from patients with PCS

Bulk RNA sequencing was performed on vastus lateralis muscle specimens obtained from all included individuals (Additional file 2). Principal component analysis resulted in a similar clustering by condition as observed in the above-mentioned muscle proteomics analysis. Although we observed enriched genes located on the Y-chromosome, corresponding to the sex-misbalance between the HDC and the two other cohorts, we did not see any sex-related influence on the PCA. Similarly, no age-related effect on the PCA was apparent (Additional file 1: Fig. S5A). When excluding all Y-Chromosome related genes, 135 genes were differentially expressed in the comparison PCS vs. HDC (*p*-value ≤ 0.001, 114 upregulated, 21 downregulated), 219 genes in the comparison PCS vs. 2BA (*p*-value ≤ 0.001, 218 upregulated, 11 downregulated) and 28 in the comparison 2BA vs HDC (*p*-value ≤ 0.001, 18 upregulated, 10 downregulated) (Additional file 1: Fig. S5B).

**Fig. 5:**
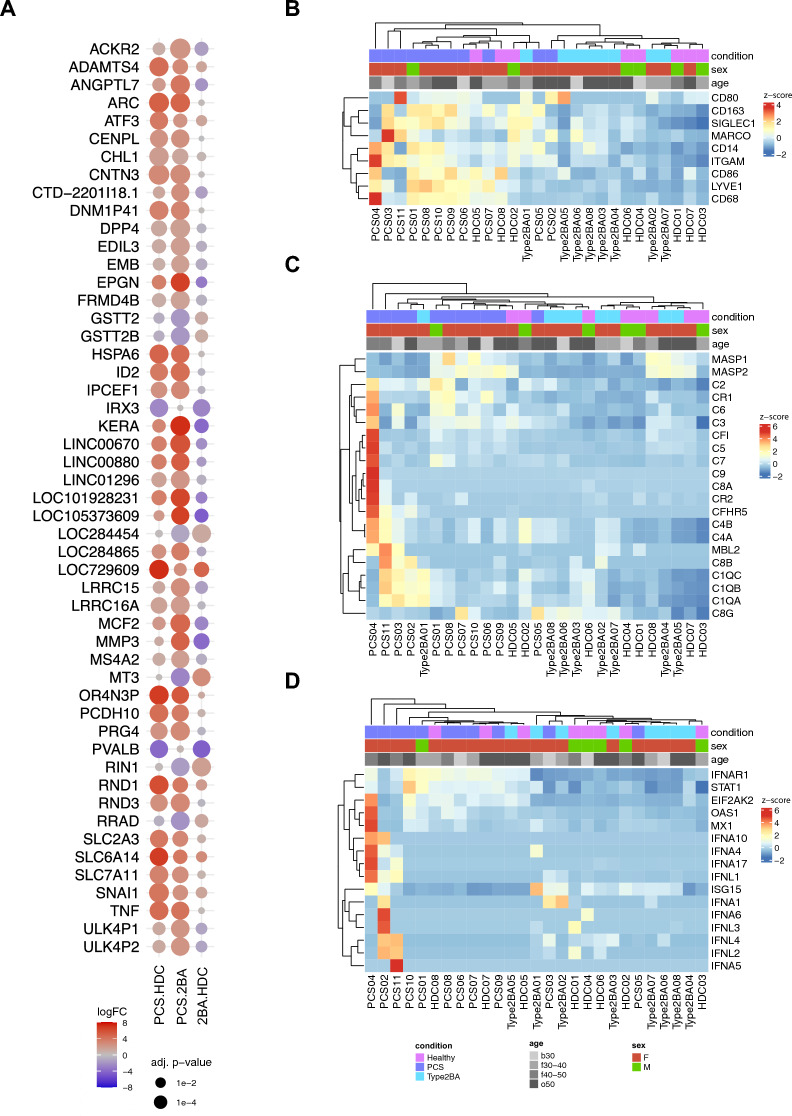
Distinct transcriptomic profiles of vastus lateralis muscles from patients with PCS. Dot plot visualization of top differentially expressed genes comparing all samples from the PCS cohort to the HDC and 2BA cohorts respectively (**A**). Dot size is proportional to the adjusted p-value. Dot color indicates logFC. Clustered heat map visualizations of macrophage marker gene expressions levels (**B**). Clustered heat map visualizations of the gene expression levels of complement pathway related genes (**C**). Clustered heat map visualizations of the gene expression levels of type I and III interferon related genes (**D**)

Among the top differentially expressed genes (*p* ≤ 0.001) were those related to extracellular matrix remodeling (*ADAMTS4, MMP3*), angiogenesis (*ANGPTL7*) and immune system regulation (*TNF*) (Fig. 5A). Of note, the patient in which we identified massive structural capillary damage (*PCS-4*) showed the highest expression levels of differentially expressed genes (Fig. 5B–D, Additional file 1: Fig. S4C).

Unbiased clustering revealed a certain heterogeneity within the PCS group, with some samples showing gene expression levels resembling those of control samples from patients with type-2b-fiber atrophy, while other samples from the PCS cohort showed clearly distinct transcriptomic signatures when compared both to the HDC and 2BA control cohort (Fig. 5B–D).

Macrophage marker genes (*CD68, SIGLEC1, CD163, LYVE1, CD86, ITGAM*) were upregulated in the PCS cohort, confirming the histological observations, albeit not reaching a *p*-value ≤ 0.001 except for CD68 in the comparison PCS vs 2BA (LogFC 0.95, *p* = 0.001) (Fig. 5B). When clustering all samples by their expression levels for complement pathway or type I and III interferon related genes respectively, many samples from patients with PCS grouped closely together and showed a distinct gene signature compared to the two control cohorts (Fig. 5C–D).

For pathway analysis, all differentially expressed genes with a *p*-value ≤ 0.01 were used. Among the top biologically relevant upregulated pathways were “*regulation of leukocyte activation*”, “*positive regulation of cell motility*”, “*vasculature development*” for the comparison PCS vs HDC and “*positive regulation of locomotion*”, “*cell–cell adhesion*”, “*inflammatory response*”, “*NABA Matrisome associated*”, “*chemotaxis*”, “*blood vessel development*”, “*extracellular matrix organization*” and “*NABA Proteoglycans*” in the comparison PCS vs 2BA. Among the top biologically relevant, downregulated pathways were “*mitochondrion organization*”, “*oxidative phosphorylation*” and other mitochondria-related pathways in the comparison PCS vs HDC and “*regulation of carbohydrate biosynthetic process*” in the comparison PCS vs 2BA (Additional file 1: Fig. S5E).

## Discussion

The individual and socioeconomic impact of post-COVID syndrome and other post-infectious syndromes such as ME/CSF is considerable [21, 48, 49]. Affected patients often face a double challenge, the one of the direct physical and mental suffering, and the one of the psychological burden of being affected by a disease with no established biomarkers and absence of clear-cut, easily observable structural alterations. Therefore, studies on the tissue level as well as translational integrative studies combining clinical observations with histopathological and molecular findings are urgently needed.

With all the tragedies related to the SARS-CoV-2 pandemic, it also offers the unique opportunity for studying post-viral syndromes in a more homogeneous manner than possible previously. To our knowledge, this is the first case–control study examining skeletal muscle tissue obtained from patients with persisting post-infectious fatigue and exercise intolerance that newly occurred after an infection with SARS-CoV-2. In one descriptive case series lacking controls, histological changes and capillary alterations were described in deltoid muscles of patients with post-COVID syndrome [50].

In the present interdisciplinary case–control cohort study, comparing patients with post-COVID syndrome to two distinct age-matched historical control cohorts, we found, on the morphological level, capillary alterations consisting of a decreased capillary-to-fiber ratio and an increased capillary basement membrane thickness. Patients with PCS showed a selective atrophy of type-2b-fibers, confirming previous reports by others [50]. Additionally, patients with PCS displayed overall smaller muscle fibers as reflected by a lower MCSFA that could not be solely explained by the selective atrophy of type-2b-fibers.

While increased numbers of CD169^+^ macrophages were apparent in close vicinity to skeletal muscle capillaries, no evidence of an overt myositis could be found in any of the included patient’s samples. Upper leg MRI also did not reveal signs of myositis, which is consistent with recently published radiological findings [51]. However, biopsies were taken almost a year after acute infection and several case reports of biopsy-proven myositis after SARS-CoV-2 have been published [52–54], and we ourselves histologically diagnosed non-specific myositis in some patients in the subacute aftermath of mild or moderate COVID (Additional file 1: Fig. S3F). Furthermore, immune-mediated myopathy in severe COVID has been well documented by two independent autopsy studies [29, 30]. We can therefore not exclude that some individuals in our cohort may have suffered from a self-limiting acute myositis, which already had resolved by the time of the biopsy.

No SARS-CoV-2 specific RNA could be detected in any of the muscle samples by ultra-sensitive qPCR, strongly arguing against an unresolved infection of skeletal muscle tissues as the cause for the patients’ symptoms.

The mere fact that the number of CD169^+^ macrophages is increased in mildly altered skeletal muscle tissue is remarkable. CD169^+^ macrophages are increased in idiopathic inflammatory myopathies [55], indicating a prominent role in type I Interferon-related immune processes [56], and recent studies emphasized their highly specific functional programs and important roles as border-associated cells at blood vessel/parenchymal interfaces [57, 58]. CD169^+^ macrophages have further been implicated in antiviral defense, being the primary cell infected and able to capture viral particles in the blood and subsequently presenting them to B cells [59]. As numbers of circulating CD169^+^ monocytes were reported to be increased in acute stages of mild COVID-19 [60], we hypothesize that they could play a key role at the myocyte/capillary interface in our cohort of patients with PCS.

With regards to the fact that most patients from the PCS cohort displayed a selective atrophy of type-2b-fibers—which constitutes a non-specific finding observed in diverse settings leading to disuse or deconditioning of a muscle—we considered the hypothesis that any of our observations could also be the consequence of reduced mobility in these patients due to their exercise intolerance, pain or to coincidental phenomena (lockdowns, stress, mood disorders), instead of being directly related to the infection or to post-infectious mechanisms. We therefore added another historical control (biopsied before 2019) cohort to the study, consisting of vastus lateralis samples obtained from patients that displayed a selective atrophy of type-2b-fibers.

On the protein level, mass spectrometry of the vastus lateralis muscle samples revealed an upregulation of basement membrane and other extracellular matrix components only when comparing the PCS samples to the HDC samples but not in comparison to the ones with type-2b-fiber atrophy. Overall, proteome differences were rather subtle, as none of the proteins was increased more than 1.5-fold. This is however not surprising, regarding the fact that histologically little pathology could be noted in the PCS samples. Also, the vastus lateralis muscle samples we used for the HDC cohort came from symptomatic individuals where biopsies were performed for diagnostic purposes, but for which routine histological examination had revealed no morphological abnormalities.

While the muscle samples from the 2BA cohort showed a wider distribution of CBM thickness—with some individuals showing similar enlargements as the PCS cohort—and higher expressions of CBM-proteins compared to the healthy control group, the mean CBM thickness was still lower in that cohort. We speculate that the 2BA cohort was more heterogeneous than the HDC one, with some of the patients possibly being affected by yet undiagnosed musculoskeletal or systemic diseases that also affected the extracellular matrix. Alternatively, increased matricellular proteins could also be a direct consequence of the selective atrophy of type-2b-fibers, for which little molecular pathomechanistic insight exist as of today. The fact that type-2b-fiber atrophy was more pronounced in the 2BA cohort than in the PCS cohort (Fig. 2B) is however an argument against a correlation between type-2b-fiber atrophy and CBM thickness. Moreover, we screened three independent bulkRNA datasets obtained from M. vastus lateralis biopsies performed before and after so-called bed rest studies. Even in this extreme form of immobility with weeklong bedrest, it appears that there is no increased expression of CBM components (collagen IV, laminins, nidogens, heparan sulfate proteoglycans) [61–63]. Another study, with only a 48 h immobilization, revealed a decreased expression of Collagen IV constituents [64]. These studies however did not include ultrastructural assessment of capillaries, limiting the comparison. Another bed rest study revealed a preserved C/F ratio and increased capillary density due to decreased cross-sectional fiber size [65]. Of note, macrophage infiltration was associated with the muscular growth phase in a rehabilitation context, rather than the hypotrophic phase of resting [66].

On the transcriptional level, unbiased analysis revealed a certain heterogeneity within the PCS cohort, but clustering based on immune cell markers, complement pathway and type I and III interferon related genes allowed a clear separation of the patient’s samples from the controls. PCS samples not only showed an upregulation of immune regulatory genes such as tumor necrosis factor alpha (*TNFA*), but also of genes related to extracellular matrix organization and cell–cell adhesion, while pathways related to oxidative phosphorylation, mitochondria and cell respiration were downregulated. This invites for the speculation that the observed morphological alterations of the capillaries (reduced C/F; thickened CBM) are indeed responsible for metabolic disturbances, possibly explaining the exercise-dependent symptomatology.

From a theoretical, physiological perspective, an increased CBM thickness results in a reduced diffusion of oxygen according to Fick's Law.^2^ Furthermore, exercise-induced increase of muscle capillarization was found to positively correlate with VO_2 max_^3^ [67], which calls for the assumption that a decreased capillarization – as observed in our cohort of PCS patients who show a decreased capillary-to-fiber-ratio – results in a lower VO_2 max_, possibly explaining the exercise-dependent symptoms.

Impaired oxygen delivery to skeletal muscles has been previously described in patients with ME/CFS [68], consisting mainly of a reduced peak oxygen uptake during physical activity [69] and oxygen therapy has been shown to improve the symptoms [70]. On the other hand, endothelial damage and capillary pathology have been extensively described in acute as well as in post-acute sequelae of SARS-CoV-2 infection in both human and animal studies [71–76]. It therefore seems plausible that a capillaropathy at least contributes to the described symptoms in a subset of patients with post-COVID syndrome.

Viral infections are well-known triggers for a multitude of autoimmune processes [77, 78]. Our findings suggest a persistent local immune system activation in subsets of patients with PCS even one year after initial infection, which in the absence of evidence for an unresolved infection and the presence of autoantibodies in some individuals from our cohort, may point towards immune system dysregulations or an autoreactivity, consistent with multiple observations in patients with acute and post-acute COVID-19 [79–85].

Mass spectrometric comparison of sera from patients with PCS to sera from individuals that fully recovered from a SARS-CoV-2-infection revealed increased abundances of proteins involved in the complement and coagulation cascade. Of note, gene expression levels of complement genes including *C1QA*, *C1QB* and *C1QC* were also increased on the tissue level in skeletal muscle specimens. An increased C1q production has been described in certain infectious and inflammatory conditions and is thought to mainly result from increased production of macrophages and dendritic cells [86].

To conclude, we found a positive correlation between morphological alterations of skeletal muscle capillaries and clinical functional scores in individuals that developed a muscular fatigue and exercise intolerance after an infection with SARS-CoV-2. This suggests but does not prove a connection between the initial viral infection and the persistent symptoms. The elevated number of CD169^+^ macrophages and distinct transcriptomic changes on the tissue level even one year after infection, together with the absence of SARS-CoV-2-specific RNA and higher levels of complement system related proteins in the serum suggest a persistently dysregulated immune system response potentially responsible for the observed microvascular alterations in our cohort of patients. Larger studies may allow to identify a *capillaropathy subset* among patients suffering from PCS, ME/CFS or other post-infectious syndromes, thus opening new doors for differential diagnosis and personalized therapies.

The fact that both of our control cohorts consisted of clinically symptomatic patients can be seen both as a strength and a weakness of the study: The weakness lies in the fact that as opposed to non-symptomatic, healthy subjects certain non-specific but pathological changes could be missed on the molecular level in our PCS cohort. The strength is that the observed differences can be attributed to the studied condition with a higher probability, as bystander effects of impaired health status resulting in a less active lifestyle will be leveled out to some degree.

Other limitations are the relatively small cohort of affected individuals and the lack muscle specimen from asymptomatic patients after a SARS-CoV-2 infection or of people with similar symptoms, but which were not infected with SARS-CoV-2.

## Materials and methods

### Study design

Nine patients who presented to the Charité outpatient clinic because of suspected post-COVID syndrome and two patients that were hospitalized at the Charité were included between June 2020 and November 2021 based on the following criteria:

1. Age >18 years
2. PCR-proven SARS-CoV-2 infection
3. Persistent muscular fatigue and post exertional malaise (PEM) first manifesting after infection with SARS-CoV-2 and lasting for at least 6 months
4. Exclusion of other causes explaining the symptoms listed under (3)
5. Approval for and absence of contraindication for vastus lateralis muscle biopsy

All patients signed informed consent before study inclusion and the study was approved by the Ethics Committee of the Charité—Universitätsmedizin Berlin (EA2/066/20 and EA2/163/17) in accordance with the 1964 Declaration of Helsinki and its later amendments.

Nine of the eleven patients were part of a larger prospective observational study [14]. These patients were seen at least once in the outpatient clinic, when a detailed clinical evaluation and neurological examination (muscle strength testing of major muscle groups, handgrip strength test, reflexes and sensory testing, 6-min-walk-test) was performed and serum samples were obtained. On at least one other occasion, study participants responded to online questionnaires (Bell and Chalder fatigue questionnaires, EQ-5D-5L) [87, 88] hosted in a secure REDCap database as previously described [14]. Proximal lower extremity MRI was performed in these patients on the same day or close to the day of the biopsy. Two other patients (*PCS-7* and *-10*) were included out of an inpatient setting based on the above-mentioned inclusion criteria. After inclusion, one of them (*PCS-7*) was diagnosed with a rheumatoid arthritis and primary biliary cholangitis. Clinical records were consulted for age, sex, preexisting medical conditions, onset and nature of acute and chronic clinical symptoms, laboratory results, therapeutic measures, and complications. All included patients were contacted by telephone beginning of December 2022 for a final follow-up evaluation, assessing the current state of perceived symptoms.

### Vastus lateralis muscle biopsy

Biopsies were taken according to standard procedures as previously described [89]. In short, after informed consent for the procedure had been granted, open biopsy of the vastus lateralis muscle was performed under local anesthesia (lidocaine 2%). After circumscribed incision of the skin with removal of a 2 × 5 mm section of the cutis, the muscle was carefully removed. A 15 × 15 mm part of the skeletal muscle, and a 5 × 10 mm part of the muscle fascia were acquired for histopathological assessment. After biopsy procedure at the *Department of Neurosurgery at Charité – Universitätsmedizin Berlin*, muscle tissue, and fascia were processed immediately at the *Department of Neuropathology, Charité – Universitätsmedizin Berlin.*

### Control cohorts

Cryopreserved skeletal muscle specimens obtained from the vastus lateralis muscle were selected based on the following inclusion criteria:

1. Age between 18 and 65 years
2. Vastus lateralis muscle biopsy prior to December 2019
3. Absence of known inflammatory disease, cancer or mitochondriopathy
4. Absence of increased creatinine kinase levels, pathological EMG, corticosteroid or other immunosuppressive therapy
5. For the healthy disease control (HDC) cohort: absence of any histopathological or immunohistochemical abnormality in the excised muscle tissue
6. For the type-2b atrophy control cohort (2BA): presence of a selective atrophy of type-2b-fibers but other than that absence of any histopathological or immunohistochemical abnormality in the excised muscle tissue

These biopsies had been performed for routine diagnostic reasons, and patients had consented to further processing of their samples for scientific purposes. We chose the term “healthy disease control (HDC)” as these patients were clinically diseased (symptoms reported in Additional file 1: Table S1) justifying a muscle biopsy, but did not show any histological abnormalities. Due to the high prevalence of women in our PCS cohort, we preferentially selected samples of women fulfilling the above-mentioned criteria. However, due to restricted numbers of available samples from women that fulfilled all our inclusion criteria, the HDC group was composed of five men and three women. The type-2b-fiber atrophy control cohort on the other hand consisted of women only.

The mean age of the HDC group was 42.6 years (SD 10.9; median 45 years), the one of the 2BA group was 44.6 years (SD 14.0; median 44.5 years).

### Magnetic resonance imaging (MRI)

MRI scans were acquired on a 3 Tesla scanner (MAGNETOM PRISMA®, Siemens, Erlangen, Germany). The subjects were examined in the supine position and feet first using a 28-channel sensitivity encoding torso array coil placed anteriorly. Total scan duration was approximately 35 min and included qualitative imaging by axial and coronal T2-weighted turbo spin echo (TSE), axial T1-weighted TSE, axial T1-weighted 3D volumetric interpolated breath-hold examination (VIBE) with Dixon fat suppression and reconstruction of in-/opposed phase, water- and fat-based images, axial 2D Spin-Echo (SE) T2 mapping, details are contained in Additional file 1: Fig. S1. The field of view (FOV) was at mid-thigh level and anatomic T1w/T2w imaging, T2 maps, and DTI were acquired using the same FOV and geometry. The mean diffusivity (MD), T1 and T2 relaxation times, and muscle quantitative fat fraction (MFF [%]) were evaluated using Visage Imaging Client (Software Release v7.1, Visage Imaging). Manual seeding of regions-of-interest (ROI) at mid-thigh level avoiding areas of fatty infiltration or vascular structures in the biceps femoris (BF), semitendinosus (ST), semimembranosus (SM), and vastus lateralis (VL) muscle was conducted by a radiologist with more than 8 years’ experience in musculoskeletal MRI, blinded to the patients’ clinical data. The MFF was calculated using axial 3D gradient echo-modified two-point Dixon-based MRI with a chemical shift-encoded reconstruction of the water and fat signal as i) SIFAT / (SIFAT + SIWATER) × 100 and reported as mean value of all pixels within the ROI.

One patient (*PCS-3*) interrupted the examination before the MD and T2 relaxation times were acquired.

### Virological analysis

Unfixed, cryopreserved muscle samples were used for detection and quantification of SARS-CoV-2 RNA by quantitative reverse transcription–polymerase chain reaction (RT-qPCR)**.** Only samples with at least two positive results were considered positive. Oligonucleotides targeting the leader transcriptional regulatory sequence and a region within the single-guide RNA encoding the SARS-CoV-2 E gene were used to detect single-guide RNA as described previously [90, 91].

Anti-SARS-CoV-2 IgG enzyme-linked immunosorbent assays with S1 and NCP domain substrate were performed in available serum samples according to the manufacturer’s instructions (Euroimmun AG®, Lübeck, Germany). In addition, electrochemiluminescence immunoassay (*ECLIA*) antigen tests were performed to detect S- and N-antigens according the manufacturer’s instructions (Elecsys®, Roche, Basel, Switzerland).

### Autoimmunity assays

Antinuclear antibody assays (HEp2 –IFT), myositis-specific autoantibodies (anti–nuclear matrix protein-2 [anti-NXP2], anti–transcriptional intermediary factor 1γ [anti-TIF1γ], anti–melanoma differentiation-associated gene 5 [anti-MDA5], anti–signal recognition particle [anti-SRP], anti-Mi2, anti-isoleucyl-transfer RNA [tRNA] synthetase [anti-OJ], anti–glycyl-tRNA synthetase [anti-EJ], anti–threonyl-tRNA synthetase [anti-PL7], anti–alanyl-tRNA synthetase [anti-PL12], anti-histidyl-tRNA synthetase [anti-Jo1], and anti–small ubiquitin-like modifier-1 activating enzyme [anti-SAE]), and myositis-associated autoantibodies (anti-Ku, anti-PM75, anti-PM100, and anti-Ro52) were performed in available serum samples according to the manufacturer’s instructions (ANA-Mosaik 1A EUROPattern and EUROLINE ANA-Profil 3 (IgG), EUROIMMUN Medizinische Labordiagnostika AG, Lübeck, Germany).

### Histology & immunohistochemistry

Unfixed biopsy specimens were snap-frozen in a container with isopentane in liquid nitrogen and stored at − 80 °C until further workup. Stainings on cryopreserved samples were performed on 7 μm thick cryomicrotome sections. Routine histological and enzymological staining (hematoxylin–eosin, Gömöri trichrome, periodic acid–Schiff, ATPases) were carried out according to standard procedures. Immunohistochemical staining was performed on a Benchmark XT autostainer (Ventana Medical Systems), as described previously [92].

For quantification of immune cell populations (CD68-, CD169-, CD206-, CD45- and CD8-positive cells, respectively) and semiquantitative scoring (degree of MHC class I & II upregulation and of type-2b-fiber atrophy), ten random fields of vision were examined independently by two experienced morphologists (W.S. and T.A.) at × 400 magnification with an Olympus BX50 microscope (Ocular WH10X-H/22). Semiquantitative scoring of type 2b fiber atrophy: 0 = no atrophic 2b fibers; 1 = atrophy of < 15% of 2b fibers; 2 = atrophy of 15–60% of 2b fibers; 3 = atrophy of > 60% of 2b fibers + many fibers < 20 µm in diameter. Positively stained immune cells were counted manually in 10 high-power fields of vision. Positive staining results with MHC class I and MHC class II were defined as a clear upregulation at the sarcolemma with capillaries and arterioles serving as internal positive controls. The following antibodies were used: MHC class I (DAKO; clone W6/32, 1:100), MHC class II (DAKO; M0775, 1:100) CD45 (DAKO; clone UCHL1, 1:100), CD68 (DAKO; clone EBM11, 1:100), CD8 (DAKO; clone C8/144B, 1:100), NKp46 (R&D Systems; clone MAB1850, 1:100), Siglec-1/CD169 (Novus Biologicals; clone HSn 7D2, 1:200), C5b-9 (DAKO/M777; clone aE11 1:100), CD206 (Abnova clone 5C11; 1:50).

### Transmission electron microscopy and capillary morphometry

Electron microscopy was performed as described previously [91]. In short, after fixation in 2.5% glutaraldehyde in 0.1 M sodium cacodylate buffer muscle samples were incubated with 1% osmium tetroxide in 0.05 M sodium cacodylate and embedded in Renlam resin after dehydration by a graded acetone series. Semithin sections of 500 nm were cut with an ultramicrotome (Ultracut E, Reichert-Jung) and a Histo Jumbo diamond knife (Diatome) and stained with toluidine blue at 80 °C. Ultrathin sections of 70 nm were cut using the same ultramicrotome and an Ultra 35° diamond knife (Diatome) and stained with uranyl citrate. Standard transmission EM was performed using a Zeiss 906 microscope in conjunction with a 2 k CCD camera (TRS).

For each patient, between 20 and 30 randomly selected capillaries from at least two different Renlam resin blocks were photographed at a final magnification of 7000 × . Blurry images with unclear basement membrane borders as well as images of abnormally large microvessels or capillaries with very high pericyte coverage were excluded from the analysis. Capillary basement membrane (CBM) thickness was measured at six distinct sites per capillary with *Image J v1.53c* (NIH, Bethesda, MD, United States), omitting areas close to profiles of pericyte processes where the CBM is irregular and usually thicker at these sites. For the evaluation of compartmental organization of capillaries, micrographs showing capillary profiles with an aspect ratio (ratio of the smallest to largest diameter) of more than 1.2 were considered too obliquely sectioned and were excluded from morphometric evaluation, as previously recommended [93].

Tablet-based image analysis (TBIA) was performed for capillary morphometry, as previously described [94]. On electron micrographs of capillary profiles, lines were drawn with a digital pen using *ImageJ* around the capillary lumen (blood:EC transition), along the abluminal EC surface (EC:BM transition), along the basement membrane (BM):endomysium transition, and around the PC surface to obtain values for areas and circumferences. Absolute arithmetic values for the lumen radius, and the EC and BM thicknesses were calculated using formulae previously described [95].

For subjective scoring of capillary alterations, images were interpreted in a blinded fashion, side-by-side by two neuropathologists (W.S. and H.H.G.) and independently by one physiologist (O.B.) with long-standing experience in ultrastructural analysis of skeletal muscle capillaries. The following scores were used per capillary: 0 =  < 4 pericyte processes; no reduplication; no ensheathment; maximum 2–3 endothelial cells; vesicles and mitochondria within normal range; 1 = single prominent pericytes; single or no ensheathment; slightly increased number and size of vesicles and mitochondria; 2 = prominent pericytes with irregular structure; ensheathment, prominent vesicles and mitochondria; 3 = same as 2 but more pronounced; 4 = loss; necrosis of endothelial cells; capillary remnants; cellular debris.

### Morphometry of light micrographs for the evaluation of capillarity

Semithin sections were prepared as described above and per patient at least 10 images at a magnification of 400 × were taken with a Keyence BZ-X810. After anonymization of the obtained images, each of these light micrographs was overlaid with a digital counting grid consisting of 10 × 10 test lines, as previously reported [96]. For calculation of the capillary-to-fiber (C/F) ratio, the number of capillary profiles and that of muscle fibers were counted within the counting grid, taking into account the so-called forbidden line rule. For an estimate of the mean cross-sectional fiber area (MCSFA), the number of test points falling on fiber profiles was divided by the total number of test points and multiplied by the total area of the counting grid (= total area of muscle fiber profiles) divided by the number of muscle fiber profiles that fell within the confines of the grid. The capillary density was determined by dividing the number of capillary profiles that fell within the boundaries of the grid by the total area of muscle fiber profiles.

### Muscle sample preparation for proteomics

Muscle specimens were lysed in 200 µl of 50 mM TEAB (pH 8.5) buffer, 5% SDS, and complete ULTRA protease inhibitor (Roche) using the Bioruptor® (Diagenode) for 10 min (30 s on, 30 s off, 10 cycles) at 4 °C. To ensure complete lysis we conducted an additional sonication step using an ultra-sonic probe (30 s, 1 s/1 s, amplitude 40%) followed by centrifugation at 4 °C and 20,000 g for 15 min. Protein concentration of the supernatant was determined by BCA assay according to the manufacturer’s protocol. Disulfide bonds were reduced by addition of 10 mM TCEP at 37 °C for 30 min, and free sulfhydryl bonds were alkylated with 15 mM IAA at room temperature (RT) in the dark for 30 min. 100 µg protein of each sample was used for proteolysis using the S-Trap protocol (Protifi) and using a protein to trypsin ratio of 20:1. The incubation time for trypsin was changed to 2 h at 47 °C.

All proteolytic digests were checked for complete digestion after desalting by using monolithic column separation (PepSwift monolithic PS-DVB PL-CAP200-PM, Dionex) on an inert Ultimate 3000 HPLC (Dionex, Germering, Germany) by direct injection of 1 μg sample. A binary gradient (solvent A: 0.1% TFA, solvent B: 0.08% TFA, 84% ACN) ranging from 5 to 12% B in 5 min and then from 12 to 50% B in 15 min at a flow rate of 2.2 μL/min and at 60 °C, was applied. UV traces were acquired at 214 nm [97].

### Proteomic analysis of muscle samples

An UltiMate 3000 RSLC nano UHPLC coupled to a QExactive HF mass spectrometer was used for the analysis of all samples, and the total amount of peptides used was always 1 µg. Samples were first transferred to a 75 µm × 2 cm, 100 Å, C18 precolumn at a flow rate of 10 µl/min for 20 min followed by separation on the 75 µm × 50 cm, 100 Å, C18 main column with a flow rate of 250 nl/min and a linear gradient composed of solution A (99.9% water, 0.1% formic acid) and solution B (84% acetonitrile, 15.9% water, 0.1% formic acid), with a pure gradient length of 120 min (3–45% solution B). The gradient was applied as follows: 3% B for 20 min, 3–35% for 120 min, followed by 3 wash steps, each reaching 95% buffer B for 3 min. After the last wash step, the instrument was equilibrated for 20 min. MS data were acquired in data independent acquisition (DIA) mode using an in-house generated spectral library for the corresponding tissue or body fluid. Each sample was mixed with an appropriate amount of iRT standard (Biognosys). Full MS scans were acquired from 300 to 1100 m/z at a resolution of 60,000 (Orbitrap) using the polysiloxane ion at 445.12002 m/z as the lock mass. The automatic gain control (AGC) was set to 3E6 and the maximum injection time was set to 20 ms. The full MS scans were followed by 23 DIA windows, each covering a range of 28 m/z with an overlap of 1 m/z, starting at 400 m/z, acquired at a resolution of 30,000 (Orbitrap) with an AGC of 3E6 and an nCE of 27 (CID).

For the analysis of samples acquired by nano-LC–MS/MS in DIA mode, the data were entered into Spectronaut software (Biognosys) and analyzed using a library-based search. The library utilized were the in-house created spectral libraries, depending on the tissue or body fluid type. The search and extraction settings were kept as default (BGS Factory settings). Human proteome data from UniProt (www.uniprot.org) with 20,374 entries were selected as the proteome background. For reliable label-free quantification, only proteins with ≥ 2 unique peptides were considered for further analysis. The average normalized abundances were determined using Spectronaut for each protein and used to determine the ratio between the patients' samples and the corresponding controls.

### Quantitative reverse transcription PCR (qRT-PCR)

Total RNA was extracted from muscle specimens using a trizol-chloroform method as described previously [92] and cDNA was synthesized using the High-Capacity cDNA Archive Kit (Applied Biosystems, Foster City, CA, USA). For qPCRs, 10 ng of cDNA was used for subsequent analysis, using an Applied Biosystems™ QuantStudio™ 6 Flex Real-Time PCR System (ThermoFischer, Waltham, MA, USA) with the following running conditions: 95 °C for 20 s, 95 °C for 1 s, 60 °C for 20 s, for 45 cycles (values above 40 cycles were defined as not expressed). All targeted transcripts were run as triplicates. For each of these runs, the reference gene *GAPDH* has been included as an internal control to normalize the relative expression of the targeted transcripts. The qPCR assay identification numbers, TaqMan® Gene Exp Assay from Life Technologies/ ThermoFisher are listed as follows: *GAPDH* Hs02786624_g1, *COL4A1* Hs00266237_m1, *COL4A2* Hs05006309_m1, *NID1* Hs00915875_m1, *NID2* Hs00201233_m1, *HSPG2* Hs01078536_m1. The ΔCT of HDCs was subtracted from the ΔCT of COVID patients muscles to determine the differences (ΔΔCT) and fold change (2^-ΔΔCT) of gene expression.

### Bulk RNA-sequencing

RNA was isolated using Trizol (ThermoFisher) and the DirectZol kit (Zymo) according to manufacturer’s instruction. Poly-A RNA sequencing libraries were then prepared using the NEBNext Ultra II Directional RNA Library Prep Kit (NEB), and sequenced to a depth of 20–30 million paired-end reads on a NovaSeq 6000 device (Illumina) with 2 × 109 sequencing length. Since the samples were sequenced together with samples containing high amounts of SARS-CoV-2 RNA on the same Novaseq sequencing lane, there were some reads aligning to the SARS-CoV-2 genome due to index hopping on the flowcell, i.e. misassignement of samples with high SARS-CoV-2 amount to the muscle samples. Due to this effect, quantification of SARS-CoV-2 RNA from sequencing is less reliable than from RT-qPCR.

### RNA sequencing analysis

Due to data protection restrictions, raw sequencing reads cannot be made readily available, however a read count table is provided as online Additional file 1: data. Raw sequencing reads were aligned to version hg19 of the human genome using hisat2 [98] with standard parameters. Read counts were quantified based on the hg19 RefSeq annotation using quasR [99]**.** Differential expression values were calculated using edgeR [100]. Further analysis was done and plots were generated using the R packages PCAtools [PCAtools: Blighe, K., Lun, A. PCAtools: everything principal components analysis. https://github.com/kevinblighe/PCAtools. R package version 2.10.0, https://github.com/kevinblighe/PCAtools], ComplexHeatmap [ComplexHeatmap: Gu Z (2022). “Complex Heatmap Visualization.” iMeta. https://doi.org/10.1002/imt2.43.], as well as packages from the Tidyverse [https://doi.org/10.21105/joss.01686]. All code is available on https://github.com/landthalerlab.

### Pathway enrichement analysis of proteomic and transcriptomic datasets

For pathway analysis of the proteomic datasets, significantly regulated proteins (*p*-value ≤ 0.05) with either positive or negative regulation (calculated fold change of ≥ 1.2 or ≤ 0.8) were included. For pathway analysis of the transcriptomic datasets, significantly regulated genes (*p*-value ≤ 0.01) with either positive or negative regulation (calculated log fold change of ≥ 0.8 or ≤ -0.8) were included using Metascape (https://metascape.org) [101].

### Statistical analysis

Statistical analyses were done and graphs created with *GraphPad Prism 9* (GraphPad Software) and *R* Software (R Core Team (2022) R: a language and environment for statistical computing. Vienna, Austria: R Foundation for Statistical Computing; 2022. Available from: https://www.R-project.org/).

Normality testing was performed (D’Agostino & Pearson, Anderson–Darling, Shapiro–Wilk and Kolmogorov–Smirnov tests) and a Gaussian distribution was confirmed for the following parameters: immune cell quantification (CD68, CD169, CD206, CD45, CD8), capillary-to-fiber-ratio, MCFSA, capillary density, CBM thickness. One-way Anova with Tukey’s multiple comparison test was used to compare differences in these parameters between the three cohorts. Data are presented as counts, percentages or means (SDs). Values were considered significant at *p* ≤ 0.05. The significance of the abundance changes in the proteomics and transcriptomic datasets were calculated with DESeq [102]. Abundance changes were considered significant at *p* ≤ 0.05 (proteomics) and at *p* ≤ 0.001 (transcriptomics). Heat map and dot plot visualizations were created with *R* Software and tables with *Excel 2016* (Microsoft).

### Supplementary Information

**Additional file 1.** Summary of clinical data.

**Additional file 2.** Bulk SEQ raw data.

## Data Availability

Any data not published within the article or at indicated online locations, can be accessed by any qualified investigator upon demand to the corresponding author.

## Footnotes

1 Bell, D. S. *The doctor’s guide to chronic fatigue syndrome: Understanding, Treating and Living with CFIDS. Boston: Da Capo Lifelong Books* (1995).

2 V'_gas_ = $D\times A\times \div \frac{\Delta P}{{\varvec{T}}}$ [V'_gas_ = *Degree of diffusion of the gas across a permeable membrane*, D = *Diffusion coefficient of the gas and the membrane*, A = *Surface of the membrane*, ΔP = *Difference in partial pressure of the gas across the membrane*, T = thickness of the membrane].

3 VO_2 max_ = maximum rate of oxygen consumption during physical exertion.
