# Supplementary material for: Post-COVID exercise intolerance is associated with capillary alterations and immune dysregulations in skeletal muscles
Source: Acta Neuropathol Commun. 2023 Dec 8;11:193. doi: 10.1186/s40478-023-01662-2 (PMC10704838; doi:10.1186/s40478-023-01662-2)
Supplement: Supplementary file 1 — Additional file 1. Summary of clinical data. [file 40478_2023_1662_MOESM1_ESM.pdf]

Supplementary Table 1

	acute COVID symptoms (<14 days)	chronic symptoms (>6 Months)	interval between acute symptoms and chronic symptoms (days)	days between acute symptom onset and biopsy (days)	comorbidities	ME-CFS (CCC)	immuno-suppressive treatment	relevant lab findings	heart MRI
PCS-1	loss of taste and smell	Muscular weakness, fatigue, PEM, dysbalance	24	452	recurring sinusitis	no	none		
PCS-2	loss of taste and smell, dyspnea, fatigue, arthralgia, cough, rhinitis, hearing impairement, gait disturbance, myalgia, sore throat, cognitive impairement, sleep disturbance, tracheobronchitis	fatigue, PEM, cognitive impairement (memory), dyspnea, burning pain with focus on joints, distal arms and legs	0	398	Lyme disease, facial paralysis, hypothyroidism	yes	Prednisolon		
PCS-3	fatigue, dyspnea, headaches, vertigo/dizziness, cough, chest pain/tightness, vomiting/nausea, numbness (legs, body)	shivering, pain (NRS 3), cramps, exercise intolerance, PEM	0	437	Asthma, endometriosis, hypothyroidism, neurodermatitis	no	none		
PCS-4	loss of taste and smell, fatigue, tachycardia, body aches, chest pain, back pain, fatigue, subfebrile temperature, rhinitis, sweating	fatigue, PEM, myalgia	0	457		yes	none	MBL-deficiency, ANA 1:1280, IgG3 subclass deficiency	05/2020:apical pericarditis 02/2021: normal appearance
PCS-5	loss of taste and smell, dyspnea, cough, fever, vertigo, myalgia, body aches	muscular weakness, myalgia, pronounced on arms, PEM	39	225	Arterial hypertension, multiple torn ligaments	no	none		diffuse fibrotic changes
PCS-6	loss of taste and smell, fatigue, mild dyspnea, chest pain/tightness, fever, sore throat, rhinitis, headaches, chills, night sweat	muscular weakness (full body), PEM	30	425	POTS	no	none		
PCS-7	fatigue, myalgia, arthralgia, joint swelling	myalgia, PEM, paresthesia, distal paresis arms, unclassified tremor, hair loss, fatigue, cognitive impairement (Concentration, memory)	31	501	Raynaud's phenomenon, primary biliary cholangitis, Polyarthritis	no	Prednisolon; azathioprin; methotrexat	anti-AMA-M2 positive	
PCS-8	headaches, cognitive impairement	Fatigue, myalgia, PEM, cognitive impairement (concentration), sleep disturbance	0	233	Arterial hypertension	no	none		
PCS-9	fatigue, headaches, myalgia/body aches, arthralgia, fever, rhinitis, cough, sore throat	Fatigue, Myalgia, PEM, cognitive impairement (concentration, memory, speech), sleep disturbances, headaches, arthralgias	13	260		yes	none	MBL-deficiency	
PCS-10	loss of taste and smell, headaches, body aches, arthralgia, myalgia, cognitive impairement	Myalgia, PEM, fatigue, hyposmia, sleep disturbances, cognitive impairement, headaches	18	339		yes	none		
PCS-11	Dyspnea requiring non-invasive highflow ventilation for 4 days, fever, cough	Persisting Dyspnea (NHYA II), PEM, exercise intolerance, fatigue, headaches, myalgia, arthralgia, heart palpitations, cognitive impairement (memory, concentration, lacking words), sleep disturbance	27	342	Class III obesity	yes	none		dilated cardiomyopathy
age (mean)	45.1								
age (median)	49.0								
HDC-1	/	Proximal muscular weakness				/	none		/
HDC-2	/	Myalgia and muscular weakness legs, intermittend muscle spasms/stiffness			WPW-Syndrome; Tachycardia, aortic aneurysm; hypercholesterolemia	/	none		/
HDC-3	/	Unclear movement disorder. Suspected mitochondriopathy was ruled out histopathologically.			tobacco consumption, unclassified tremor	/	none		/
HDC-4	/	Muscle hypertrophy right leg of unknown origin; suspected hereditary muscle disease was ruled out				/	none		/
HDC-5	/	Proximally pronounced progressive muscular weakness and myalgia			Depression, Neurodermatitis, Spondylarthritis	/	none		/
HDC-6	/	Exercicse intolerance, Post-exertional malaise; muscle cramps and stiffness				/	none		/
HDC-7	/	General muscular weakness; unilateral ptosis			Myocarditis (in teenage years)	/	none	ANA 1:160; anti-SOX-1 antibodies	/
HDC-8	/	Proximal myalgia and muscular weakness				/	none		/
age (mean)	42.6								
age (median)	45.0								
2BA-1	/	Focal epilepsia, progressive global cerebral atrophy. Suspected mitochondriopathy was ruled out histopathologically.				/	none	ANA 1:160	/
2BA-2	/	Proximal muscular weakness			Arterial hypertension, smoking	/	none		/
2BA-3	/	Exercise-dependent myalgia				/	none		/
2BA-4	/	Exercise-dependent muscle weakness. Suspected mitochondriopathy was ruled out histopathologically.			arterial hypertension, hypercholesterolemia, psoriatic arthritis, epilepsia	/	none		/
2BA-5	/	Muscular weakness (arms and legs)			arterial hypertension, migraine,	/	none		/
2BA-6	/	proximal muscular weakness (legs); myalgia				/	none		/
2BA-7	/	Exercise-dependent proximal myalgia (legs)			arterial hypertension	/	none		/
2BA-8	/	Myalgia and proximal weakness (legs)			tobacco consumption, peripheral artery disease, aortic aneurysm	/	none		/
age (mean)	44.6								
age (median)	44.5								

Abbreviations: PCS = Post-COVID syndrome; PEM = post-exertional malaise; NRS= numeric rating scale; NHYA = New York Heart Association Functional Classification of heart insufficiency; ME-CFS = Myalgic encephalomyelitis / chronic fatigue syndrome; CCC= Canadian consensus criteria; POTS = Postural orthostatic tachycardia syndrome; MBL-deficiency= Mannan-binding lectin deficiency; ANA = antinuclear antibodies; anti-AMA-A2 = Anti-mitochondrial antibody; WPW = Wolff-Parkinson-White Syndrome.

Fig. S1

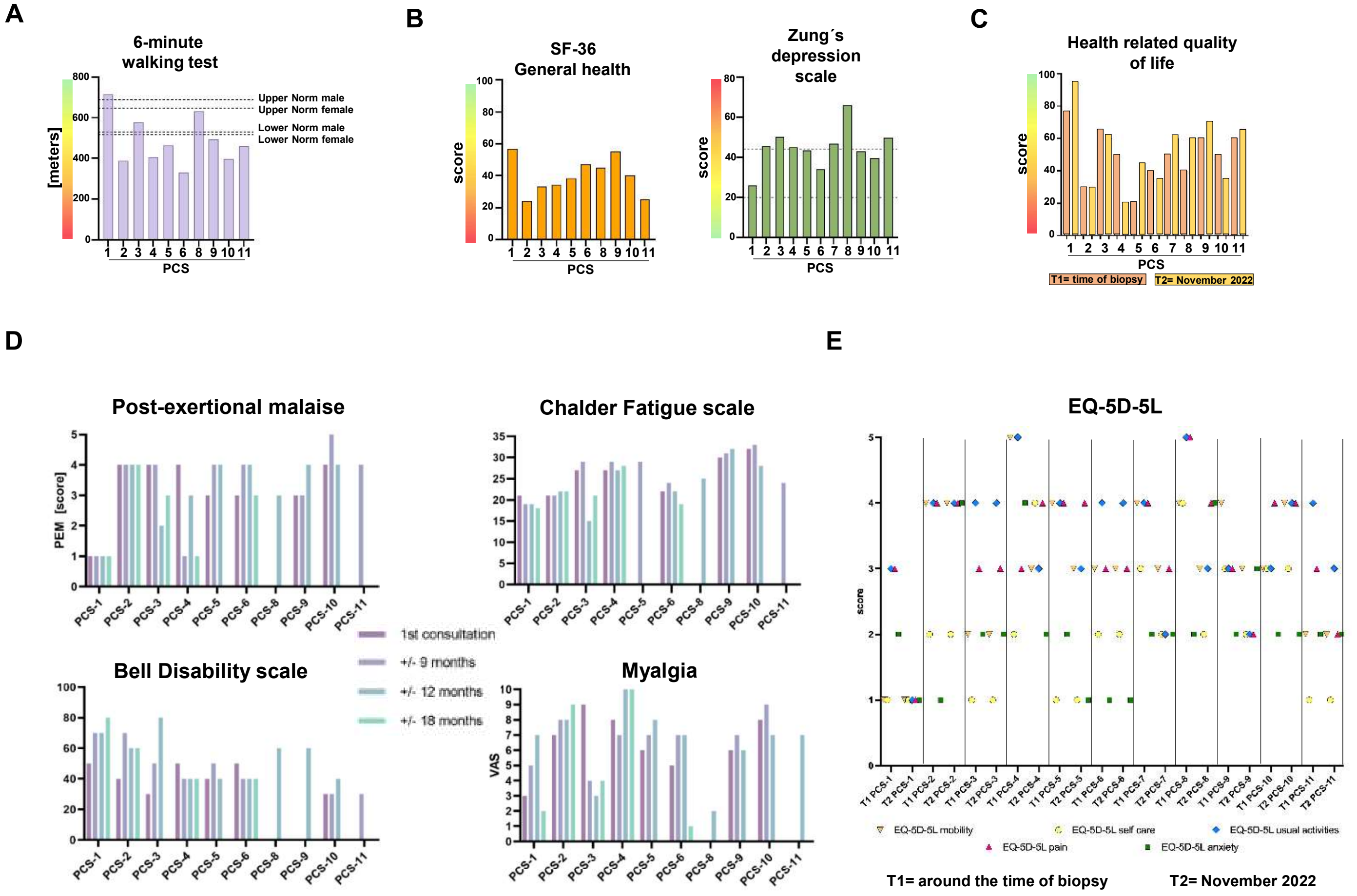

F

	Euroimmun ELISA			Roche Elecsys				
	Anti-S1 IgG ELISA OD ratio	S1 ELISA IgG	Anti-NCP IgG ELISA OD ratio	N ELISA IgG result	Anti-N value	Elecsys N	Anti-S (U/ml)	Elecsys S
PCS-1	1.47	reactive	0.21	negative	1.83	reactive	133.5	reactive
PCS-2	2.33	reactive	0.28	negative	29.69	reactive	154	reactive
PCS-3	6.21	reactive	0.55	negative	23.46	reactive	>250	reactive
PCS-4	11.34	reactive	0.08	negative	0.747	negative	>250	reactive
PCS-5	>12	reactive	0.59	negative	32.16	reactive	>250	reactive
PCS-6	>12	reactive	0.44	negative	22.9	reactive	>250	reactive
PCS-7	6.38	reactive	no data	no data	no data	reactive	no data	no data
PCS-8	8.88	reactive	0.13	negative	0.792	negative	>250	reactive
PCS-9	>12	reactive	0.64	negative	39.76	reactive	>250	reactive
PCS-10	2.09	reactive	0.13	negative	5.59	reactive	148.4	reactive
PCS-11	11.45	reactive	3.15	reactive	39.7	reactive	>250	reactive

G

Sequence	Plane	FOV (mm2)	Voxel size (mm3)	Slices	Flip angle (°)	TR (ms)	TE (ΔTE) (ms)	Additional information
DTI	axial	450x450	1.7x1.7x3.0	72	90	9100	47	b-value (s/mm2): b1=0, b2=500; NSA: b1=2, b2=3; NDGD: 20
T2 TSE stir	axial	420x420	0.8x0.8x4.0	79	150	6940	84	
T2 TSE stir	coronal	460x460	0.6x0.6x4.0	35	130	3120	38	
T1 TSE	axial	420x420	0.8x0.8x4.0	79	150	907	10	
3D T1 vibe Dixon	axial	380x380	1.2x1.2x3.0	73	9	4	1.29 (2.52)	
T2 mapping	axial	380x380	1.0x1.0x3.0	25	180	2030	TE 1 13.8; TE 2 27.6; TE 3 41.4; TE 4 55.2; TE 5 69.0	Distance factor 20%, NSA 1

H

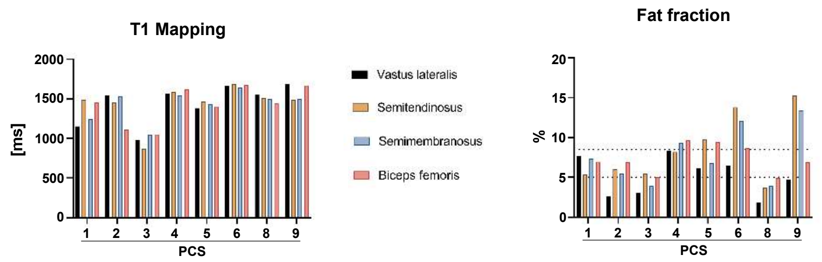

Fig. S2

PCS-1

Antigen	Intensität	Klasse	o	(+)	+	++	+++
Mi-2alpha (Mi-2a)	4	o					
Mi-2beta (Mi-2b)	4	o					
TIF1gamma (TIF1g)	1	o					
MDA5 (MDA5)	4	o					
NXP2 (NXP2)	3	o					
SAE1 (SAE1)	3	o					
Ku (Ku)	2	o					
PM-Scl100 (PM100)	3	o					
PM-Scl75 (PM75)	3	o					
Jo-1 (Jo-1)	1	o					
SRP (SRP)	8	(+)					
PL-7 (PL-7)	4	o					
PL-12 (PL-12)	3	o					
EJ (EJ)	3	o					
OJ (OJ)	3	o					
Ro52 (Ro52)	1	o					

PCS-2

Antigen	Intensität	Klasse	o	(+)	+	++	+++
Mi-2alpha (Mi-2a)	2	o					
Mi-2beta (Mi-2b)	3	o					
TIF1gamma (TIF1g)	0	o					
MDA5 (MDA5)	3	o					
NXP2 (NXP2)	3	o					
SAE1 (SAE1)	2	o					
Ku (Ku)	2	o					
PM-Scl100 (PM100)	0	o					
PM-Scl75 (PM75)	2	o					
Jo-1 (Jo-1)	3	o					
SRP (SRP)	3	o					
PL-7 (PL-7)	3	o					
PL-12 (PL-12)	1	o					
EJ (EJ)	2	o					
OJ (OJ)	1	o					
Ro52 (Ro52)	4	o					

PCS-3

Antigen	Intensität	Klasse	o	(+)	+	++	+++
Mi-2alpha (Mi-2a)	4	o					
Mi-2beta (Mi-2b)	3	o					
TIF1gamma (TIF1g)	1	o					
MDA5 (MDA5)	3	o					
NXP2 (NXP2)	3	o					
SAE1 (SAE1)	3	o					
Ku (Ku)	4	o					
PM-Scl100 (PM100)	2	o					
PM-Scl75 (PM75)	4	o					
Jo-1 (Jo-1)	1	o					
SRP (SRP)	13	(+)					
PL-7 (PL-7)	3	o					
PL-12 (PL-12)	7	o					
EJ (EJ)	2	o					
OJ (OJ)	10	(+)					
Ro52 (Ro52)	2	o					

PCS-4

Antigen	Intensität	Klasse	o	(+)	+	++	+++
Mi-2alpha (Mi-2a)	4	o					
Mi-2beta (Mi-2b)	4	o					
TIF1gamma (TIF1g)	1	o					
MDA5 (MDA5)	4	o					
NXP2 (NXP2)	5	o					
SAE1 (SAE1)	3	o					
Ku (Ku)	2	o					
PM-Scl100 (PM100)	6	o					
PM-Scl75 (PM75)	7	o					
Jo-1 (Jo-1)	2	o					
SRP (SRP)	6	o					
PL-7 (PL-7)	4	o					
PL-12 (PL-12)	2	o					
EJ (EJ)	5	o					
OJ (OJ)	2	o					
Ro52 (Ro52)	0	o					

PCS-5

Antigen	Intensität	Klasse	o	(+)	+	++	+++
Mi-2alpha (Mi-2a)	4	o					
Mi-2beta (Mi-2b)	1	o					
TIF1gamma (TIF1g)	0	o					
MDA5 (MDA5)	6	o					
NXP2 (NXP2)	3	o					
SAE1 (SAE1)	5	o					
Ku (Ku)	1	o					
PM-Scl100 (PM100)	23	+					
PM-Scl75 (PM75)	3	o					
Jo-1 (Jo-1)	2	o					
SRP (SRP)	5	o					
PL-7 (PL-7)	16	+					
PL-12 (PL-12)	3	o					
EJ (EJ)	4	o					
OJ (OJ)	4	o					
Ro52 (Ro52)	2	o					

PCS-6

Antigen	Intensität	Klasse	o	(+)	+	++	+++
Mi-2alpha (Mi-2a)	4	o					
Mi-2beta (Mi-2b)	1	o					
TIF1gamma (TIF1g)	0	o					
MDA5 (MDA5)	6	o					
NXP2 (NXP2)	3	o					
SAE1 (SAE1)	5	o					
Ku (Ku)	1	o					
PM-Scl100 (PM100)	23	+					
PM-Scl75 (PM75)	3	o					
Jo-1 (Jo-1)	2	o					
SRP (SRP)	5	o					
PL-7 (PL-7)	16	+					
PL-12 (PL-12)	3	o					
EJ (EJ)	4	o					
OJ (OJ)	4	o					
Ro52 (Ro52)	2	o					

PCS-8

Antigen	Intensität	Klasse	o	(+)	+	++	+++
Mi-2alpha (Mi-2a)	2	o					
Mi-2beta (Mi-2b)	3	o					
TIF1gamma (TIF1g)	0	o					
MDA5 (MDA5)	5	o					
NXP2 (NXP2)	2	o					
SAE1 (SAE1)	4	o					
Ku (Ku)	2	o					
PM-Scl100 (PM100)	0	o					
PM-Scl75 (PM75)	4	o					
Jo-1 (Jo-1)	1	o					
SRP (SRP)	3	o					
PL-7 (PL-7)	2	o					
PL-12 (PL-12)	2	o					
EJ (EJ)	2	o					
OJ (OJ)	3	o					
Ro52 (Ro52)	1	o					

PCS-9

Antigen	Intensität	Klasse	o	(+)	+	++	+++
Mi-2alpha (Mi-2a)	6	o					
Mi-2beta (Mi-2b)	2	o					
TIF1gamma (TIF1g)	1	o					
MDA5 (MDA5)	4	o					
NXP2 (NXP2)	3	o					
SAE1 (SAE1)	2	o					
Ku (Ku)	4	o					
PM-Scl100 (PM100)	2	o					
PM-Scl75 (PM75)	18	+					
Jo-1 (Jo-1)	1	o					
SRP (SRP)	7	o					
PL-7 (PL-7)	3	o					
PL-12 (PL-12)	28	+					
EJ (EJ)	1	o					
OJ (OJ)	5	o					
Ro52 (Ro52)	3	o					

PCS-10

Antigen	Intensität	Klasse	o	(+)	+	++	+++
Mi-2alpha (Mi-2a)	3	o					
Mi-2beta (Mi-2b)	2	o					
TIF1gamma (TIF1g)	1	o					
MDA5 (MDA5)	5	o					
NXP2 (NXP2)	5	o					
SAE1 (SAE1)	2	o					
Ku (Ku)	1	o					
PM-Scl100 (PM100)	1	o					
PM-Scl75 (PM75)	1	o					
Jo-1 (Jo-1)	1	o					
SRP (SRP)	5	o					
PL-7 (PL-7)	8	(+)					
PL-12 (PL-12)	2	o					
EJ (EJ)	2	o					
OJ (OJ)	2	o					
Ro52 (Ro52)	2	o					

Fig. S3

A Type 2b atrophy fiber score

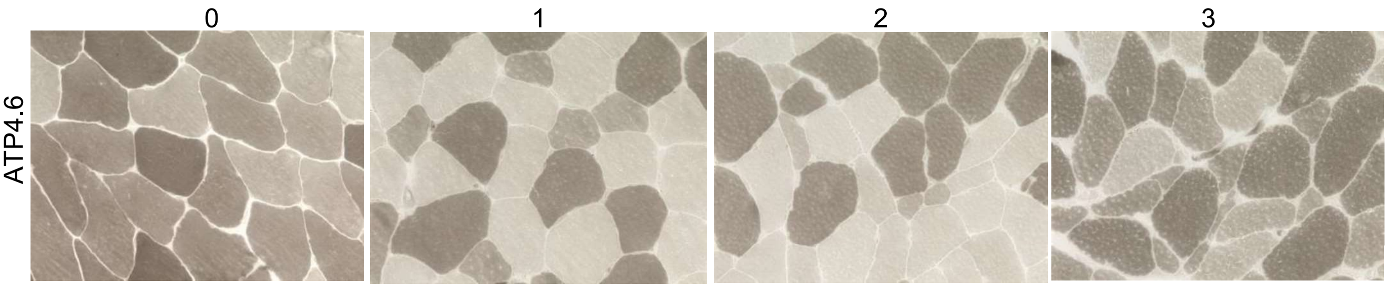

B

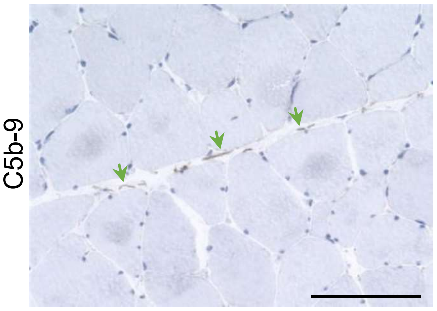

PCS-11

C

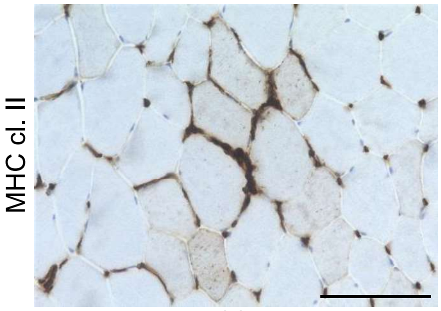

PCS-3

D

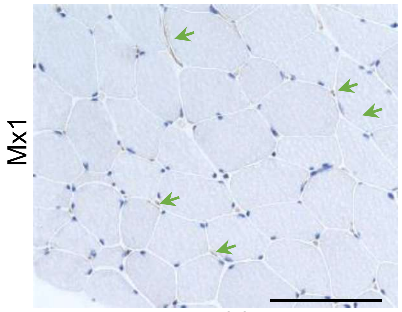

PCS-6

E

	FSV	groups	Typ 2b atrophy (score)	Type 1 Fibers (%)	Type 2 Fibers (%)	necrotic fibers	C5b-9	MHC cl. I	MHC cl. II	MxA	CD68+	CD45+	CD8+	CD169+	CD206+
HDC-1	-	-	0	80	20	0	-	1	0	-	20	1	2	4	49
HDC-2	-	-	0	50	50	0	-	0	0	-	43	22	3	13	36
HDC-3	-	-	0	75	25	0	-	0	0	-	23	14	2	20	30
HDC-4	-	-	0	45	55	0	-	0	0	-	28	8	2	8	41
HDC-5	-	-	0	45	55	0	-	1	0	-	34	5	0	18	3
HDC-6	(+)	-	0	70	30	0	-	0	0	-	52	8	1	33	28
HDC-7	(+)	-	0	70	30	0	-	0	0	-	16	5	0	13	16
HDC-8	-	-	0	60	40	0	-	0	0	-	38	7	1	28	45
mean				62	38						32	9	1	17	31
SD				14	14						12	6	1	10	15
2BA-1	(+)	-	2	30	70	0	-	0	-	-	63	25	2	42	62
2BA-2	+	-	3	50	50	0	-	1	-	-	33	14	1	35	38
2BA-3	+	-	2	25	75	0	-	0	-	-	42	5	1	15	24
2BA-4	+	-	2	75	25	0	-	0	-	-	18	6	0	13	38
2BA-5	(+)	-	2	45	55	0	-	0	-	-	37	8	1	18	40
2BA-6	(+)	-	2	70	30	0	-	0	-	-	31	4	0	13	21
2BA-7	(+)	-	2	55	45	0	-	0	-	-	12	3	0	14	15
2BA-8	(+)	-	1	60	40	0	-	0	-	-	32	15	1	24	29
mean				51	49						34	10	1	22	33
SD				18	18						15	8	1	11	15
PCS-1	+	-	1	60	40	0	-	0	-	-	71	19	0	59	49
PCS-2	+	-	2	60	40	0	-	0	-	-	60	12	1	28	46
PCS-3	+	-	1	50	50	0	-	2	+	-	60	15	7	49	58
PCS-4	+	-	1	70	30	1	-	0	-	(+)	82	15	0	61	12
PCS-5	+	-	2	70	30	0	-	0	-	-	32	23	0	24	19
PCS-6	+	(+)	2	50	50	0	-	1	-	+ (cap)	70	19	0	49	12
PCS-7	+	-	1	70	30	0	-	1	-	-	55	13	0	45	20
PCS-8	(+)	-	0	60	40	0	-	0	-	-	38	9	1	29	54
PCS-9	+	-	0	75	25	0	-	1	-	-	47	11	3	29	17
PCS-10	+	-	3	50	50	0	-	2	(+)	-	47	12	4	36	61
PCS-11	+	-	2	80	20	0	+ (cap)	0	-	-	73	10	4	44	44
mean				63	37						58	14	2	41	36
SD				11	11						16	4	2	13	20

FSV (Fiber size variation)
- \- physiological
- (+) mildly increased
- \+ increased

Typ 2b atrophy score
- 0 no atrophy
- 1 <15% of type 2b fibers
- 2 15-60% of type 2b fibers
- 3 >60% of type 2b fibers

MHC cl. I & II score
- 0 no positive fibers
- 1 <20%
- 2 20-50%
- 3 >50%

cap = capillary expression only

F

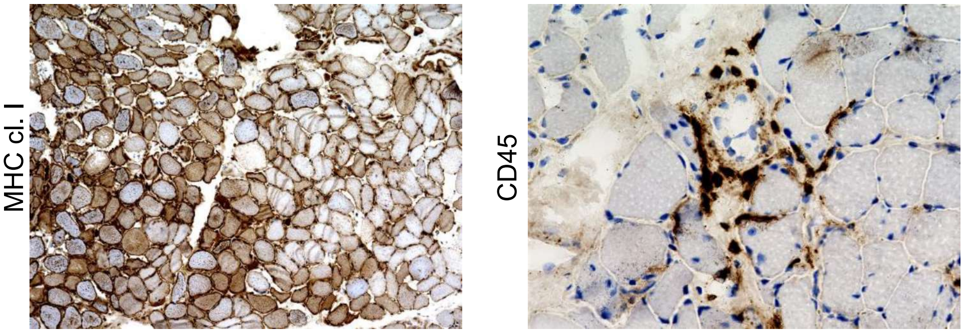

Fig. S4

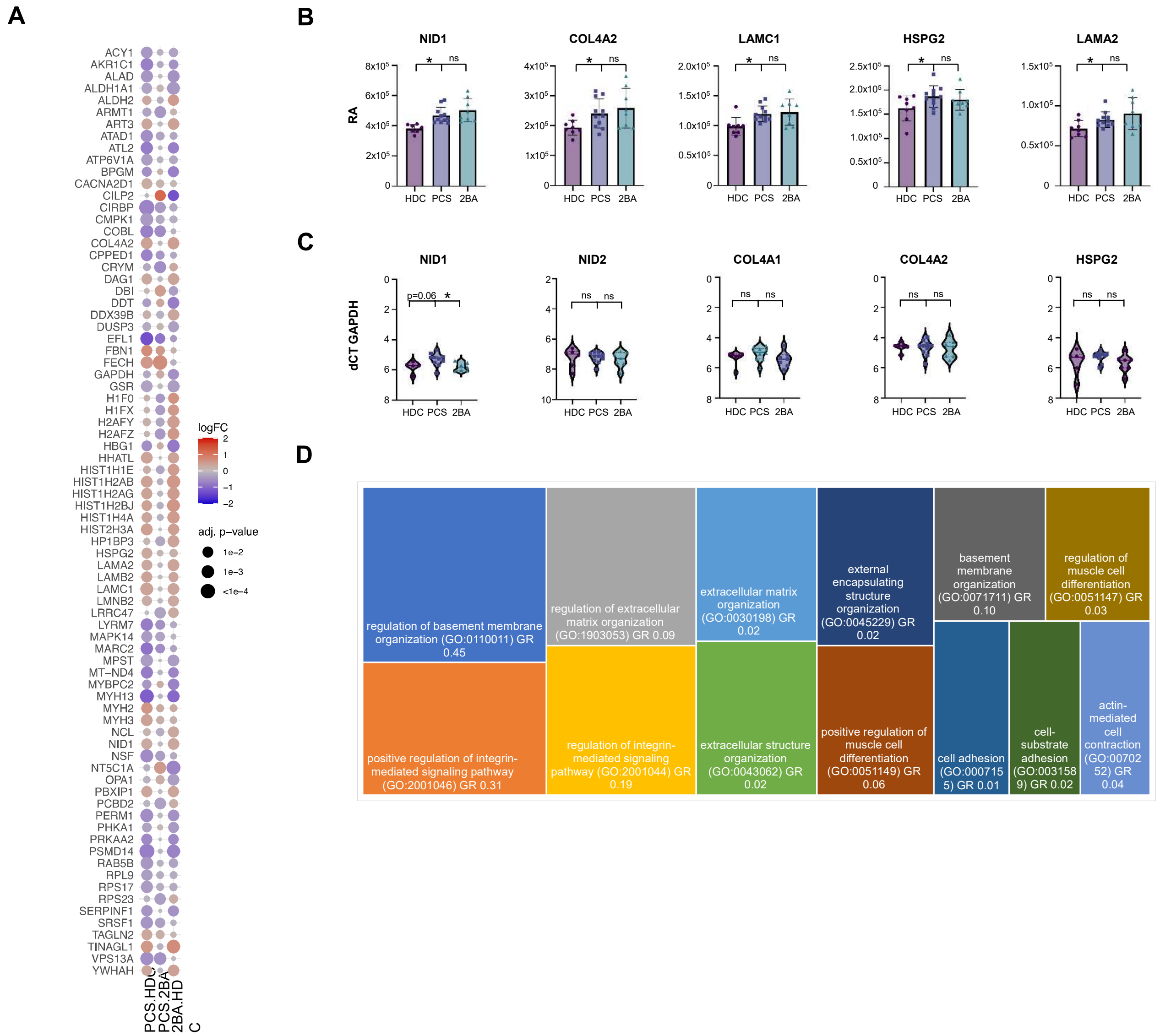
A
ACY1
AKR1C1
ALAD
ALDH1A1
ALDH2
ARMT1
ART3
ATAD1
ATL2
ATP6V1A
BPGM
CACNA2D1
CILP2
CIRBP
CMPK1
COBL
COL4A2
CPPED1
CRYM
DAG1
DBI
DDT
DDX39B
DUSP3
EFL1
FBN1
FECH
GAPDH
GSR
H1F0
H1FX
H2AFY
H2AFZ
HBG1
HHATL
HIST1H1E
HIST1H2AB
HIST1H2AG
HIST1H2BJ
HIST1H4A
HIST2H3A
HP1BP3
HSPG2
LAMA2
LAMB2
LAMC1
LMNB2
LRRC47
LYRM7
MAPK14
MARC2
MPST
MT−ND4
MYBPC2
MYH13
MYH2
MYH3
NCL
NID1
NSF
NT5C1A
OPA1
PBXIP1
PCBD2
PERM1
PHKA1
PRKAA2
PSMD14
RAB5B
RPL9
RPS17
RPS23
SERPINF1
SRSF1
TAGLN2
TINAGL1
VPS13A
YWHAH
PCS.HDC
PCS.2BA
2BA.HDC
logFC
2
1
0
−1
−2
adj. p−value
1e−2
1e−3
<1e−4
B
RA
NID1
COL4A2
LAMC1
HSPG2
LAMA2
HDC PCS 2BA
C
dCT GAPDH
NID1
p=0.06
NID2
COL4A1
COL4A2
HSPG2
HDC PCS 2BA
D
regulation of basement membrane organization (GO:0110011) GR 0.45
regulation of extracellular matrix organization (GO:1903053) GR 0.09
extracellular matrix organization (GO:0030198) GR 0.02
external encapsulating structure organization (GO:0045229) GR 0.02
basement membrane organization (GO:0071711) GR 0.10
regulation of muscle cell differentiation (GO:0051147) GR 0.03
positive regulation of integrin-mediated signaling pathway (GO:2001046) GR 0.31
regulation of integrin-mediated signaling pathway (GO:2001044) GR 0.19
extracellular structure organization (GO:0043062) GR 0.02
positive regulation of muscle cell differentiation (GO:0051149) GR 0.06
cell adhesion (GO:0007155) GR 0.01
cell-substrate adhesion (GO:0031589) GR 0.02
actin-mediated cell contraction (GO:0070252) GR 0.04

Fig. S5

A

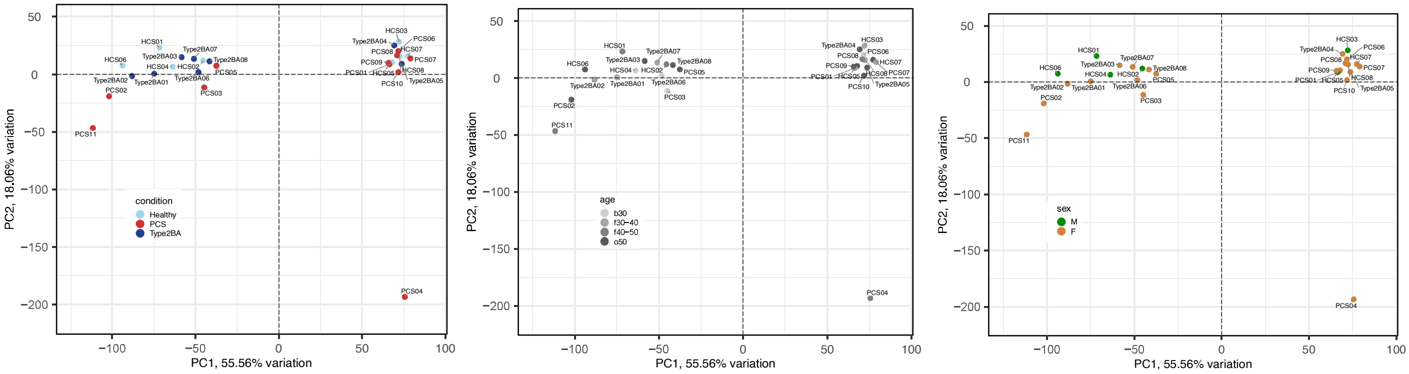

B

Number of differentially expressed genes (p≤0.001)			
	PCS vs HDS	PCS vs 2BA	2BA vs HDC
all samples	36	76	37
all samples without Y-Chromosome genes	135	219	28
all samples without PCS-4 and without Y-Chromosome genes	38	103	39
**Number of upregulated genes (p≤0.001)**			
	PCS vs HDS	PCS vs 2BA	2BA vs HDC
all samples	23	56	21
all samples without Y-Chromosome genes	114	218	18
all samples without PCS-4 and without Y-Chromosome genes	23	83	27
**Number of downregulated genes (p≤0.001)**			
	PCS vs HDS	PCS vs 2BA	2BA vs HDC
all samples	13	20	16
all samples without Y-Chromosome genes	21	11	10
all samples without PCS-4 and without Y-Chromosome genes	15	20	12

C

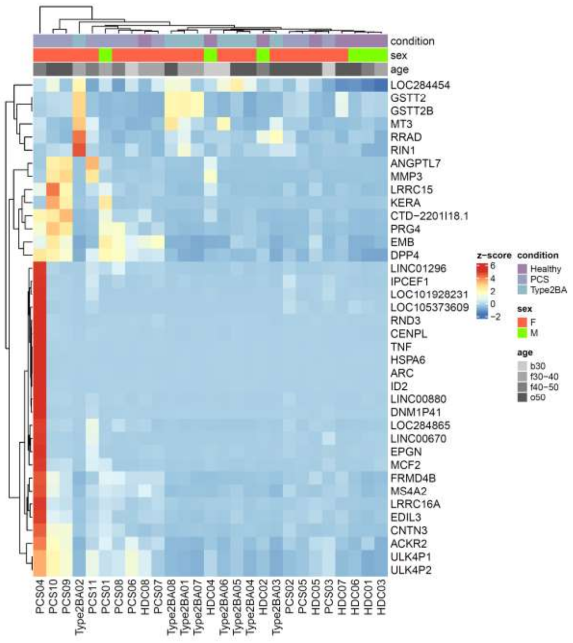

D

Top DEGs without PCS-4

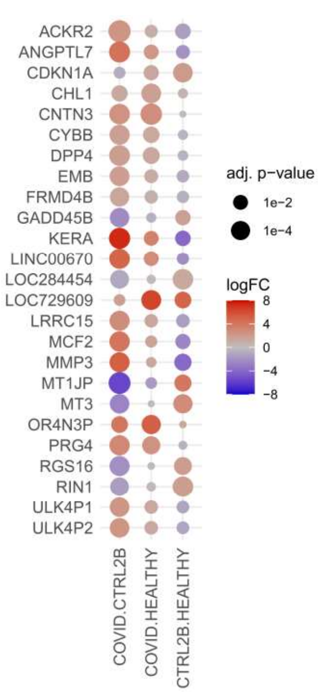

E

PCS vs HDC

upregulated pathways

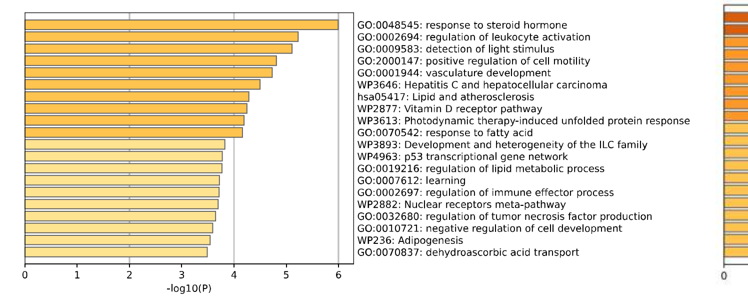

PCS vs 2BA

upregulated pathways

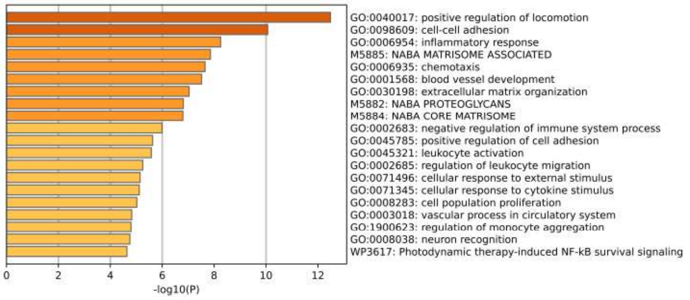

PCS vs HDC

downregulated pathways

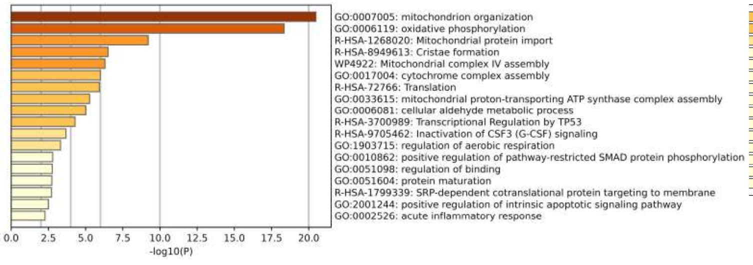

PCS vs 2BA

downregulated pathways

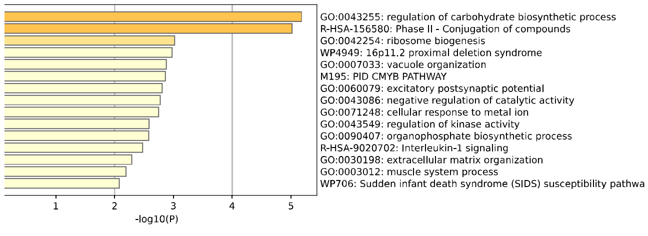

Fig. S6

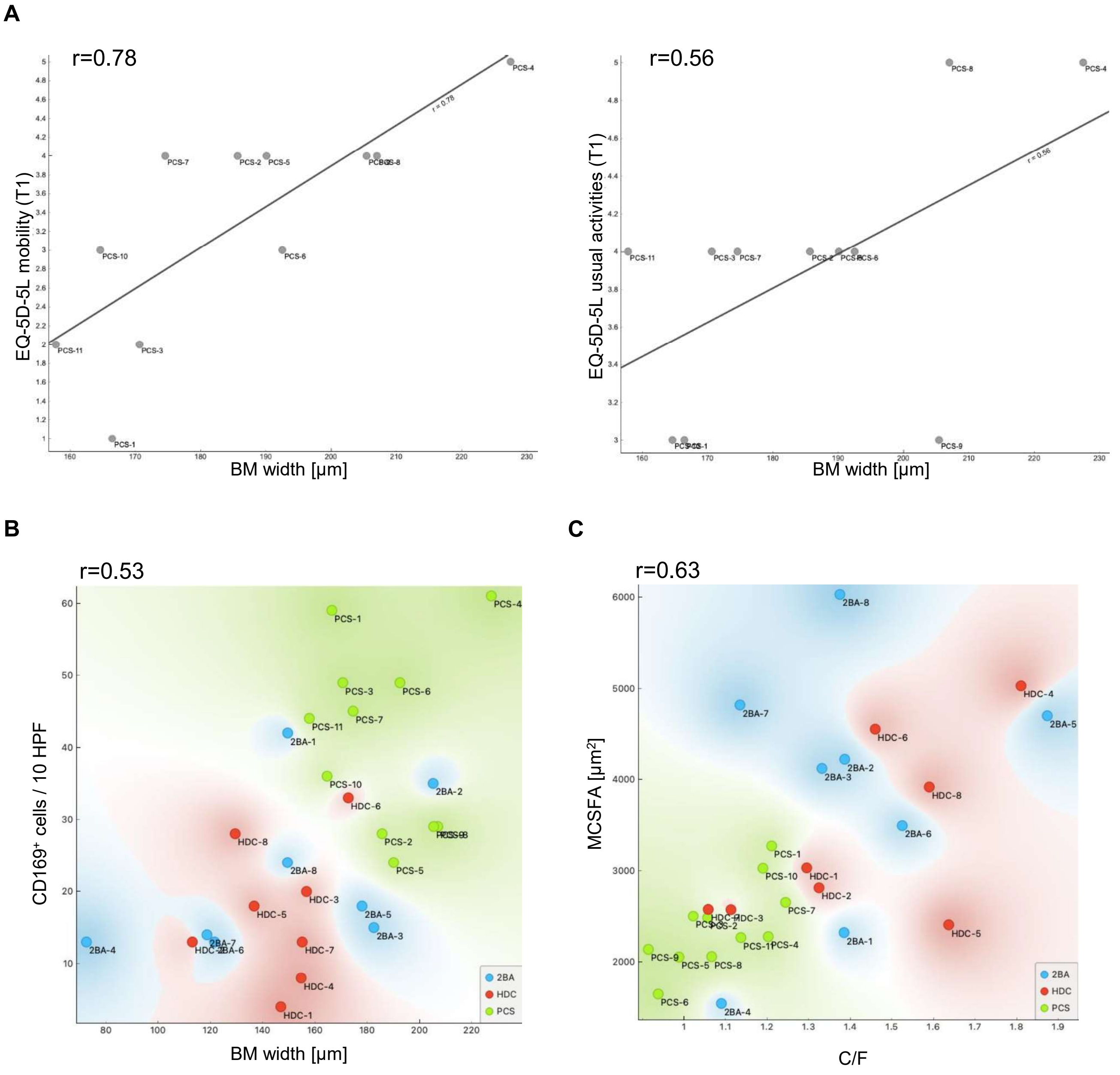

D

Pearson correlation

**1**	-0.313	Lumen radius [µm]	age
**2**	-0.224	Cap radius [µm]	age
**3**	+0.176	BM [µm]	age
**4**	+0.160	EC-thickness [µm]	age
**5**	-0.157	MHC Cl I [score]	age
**6**	-0.154	CD8 [cells/10HPF]	age
**7**	-0.139	CD169 [cells/10HPF]	age
**8**	-0.130	CD206 [cells/10HPF]	age
**9**	-0.093	C/F	age
**10**	-0.035	CD68 [cells/10HPF]	age
**11**	-0.027	CD45 [cells/10HPF]	age
**12**	-0.027	PC Coverage [%]	age
**13**	+0.016	MCSFA [µm2]	age
**14**	+0.013	2B atrophy [score]	age
**15**	+0.009	Capillary density [mm2]	age
